# Isotopic evaluation of the National Water Model reveals missing agricultural irrigation contributions to streamflow across the western United States

**DOI:** 10.5194/hess-28-2895-2024

**Published:** 2024-07-04

**Authors:** Annie L. Putman, Patrick C. Longley, Morgan C. McDonnell, James Reddy, Michelle Katoski, Olivia L. Miller, J. Renée Brooks

**Affiliations:** 1 Utah Water Science Center, US Geological Survey, Salt Lake City, Utah, USA; 2 Colorado Water Science Center, US Geological Survey, Grand Junction, Colorado, USA; 3 New York Water Science Center, US Geological Survey, Ithaca, New York, USA; 4 Maryland–Delaware Water Science Center, US Geological Survey, Baltimore, Maryland, USA; 5 Pacific Ecological Systems Division, US Environmental Protection Agency, Corvallis, Oregon, USA

## Abstract

The National Water Model (NWM) provides critical analyses and projections of streamflow that support water management decisions. However, the NWM performs poorly in lower-elevation rivers of the western United States (US). The accuracy of the NWM depends on the fidelity of the model inputs and the representation and calibration of model processes and water sources. To evaluate the NWM performance in the western US, we compared observations of river water isotope ratios (O18∕O16 and H2∕H1 expressed in δ notation) to NWM-flux-estimated (model) river reach isotope ratios. The modeled estimates were calculated from long-term (2000–2019) mean summer (June, July, and August) NWM hydrologic fluxes and gridded isotope ratios using a mass balance approach. The observational dataset comprised 4503 in-stream water isotope observations in 877 reaches across 5 basins. A simple regression between observed and modeled isotope ratios explained 57.9 % (δO18) and 67.1 % (δH2) of variance, although observations were 0.5 *‰* (δO18) and 4.8 *‰* (δH2) higher, on average, than mass balance estimates. The unexplained variance suggest that the NWM does not include all relevant water fluxes to rivers. To infer possible missing water fluxes, we evaluated patterns in observation–model differences using δOdiff18 (δOobs18−δOmod18) and $d_{\text{diff}}$ (δHdiff2−8⋅δOdiff18). We detected evidence of evaporation in observations but not model estimates (negative $d_{\text{diff}}$ and positive δOdiff18) at lower-elevation, higher-stream-order, arid sites. The catchment actual-evaporation-to-precipitation ratio, the fraction of streamflow estimated to be derived from agricultural irrigation, and whether a site was reservoir-affected were all significant predictors of $d_{\text{diff}}$ in a linear mixed-effects model, with up to 15.2 % of variance explained by fixed effects. This finding is supported by seasonal patterns, groundwater levels, and isotope ratios, and it suggests the importance of including irrigation return flows to rivers, especially in lower-elevation, higher-stream-order, arid rivers of the western US.

## 1 Introduction

The western United States (US) is experiencing multi-decadal drought (Williams et al., 2022) and declining streamflows (Milly and Dunne, 2020). Major rivers are running dry (Kornfield, 2022), lakes are shrinking (Ramirez, 2022; Fergus et al., 2020, 2022), and water users are experiencing shortages and cuts (Bureau of Reclamation, Department of the Interior, 2022). These decreases in streamflow and groundwater fluxes are projected to continue in coming years (Miller et al., 2021a, b), with projected decreases in snowpack (Mote et al., 2021; Siirila-Woodburn et al., 2021) and increases in temperatures (Hicke et al., 2022). Under drought and snow drought stress as well as changing wintertime precipitation patterns, river flows may become more difficult to forecast (Hammond and Kampf, 2020; Siirila-Woodburn et al., 2021). Thus, with decreasing water availability, water managers and other stakeholders tasked with managing and responding to current and future water supply increasingly depend on accurate streamflow predictions.

Fully routed, high-spatiotemporal-resolution streamflow models – like the National Oceanic and Atmospheric Administration’s National Water Model (NWM), which is an application of the Weather Research and Forecasting (WRF) Hydro model (Gochis et al., 2018) – provide short- and medium-term streamflow prediction in the US as well as analyses of past stream discharge at ungauged locations. The accurate, detailed, frequent results from the NWM may be used by emergency managers, reservoir operators, floodplain managers, and farmers to aid in water use decision-making and flood or pollution risk evaluation. The accuracy of predictions and current snapshots produced by the model depend on (1) inclusion and faithful representation of relevant water sources and hydrologic processes, (2) appropriate calibration of parameter estimations, and (3) the fidelity of the model inputs.

With respect to the faithful representation of water sources, the major water sources to streams in the mountainous west include two broad water flux categories: runoff (which is also called “quickflow” and may comprise surface or subsurface waters) and groundwater discharge (also called “baseflow”). Runoff during the summer comes from late-season snowmelt, rain, and irrigation water. Groundwater discharge comes from shallow or deep in-ground water, typically recharged at high elevation by snowmelt. Rivers in the west derive the majority of their water from springtime melt of the high-elevation wintertime snowpack (Li et al., 2017; Hammond et al., 2023), whereas little water is contributed to streams at lower elevations where there is minimal snowpack (Miller et al., 2021b). Some of the meltwater enters streams as surface runoff during late-spring and summer, while the remainder recharges shallow and deep groundwater and, later in the season or in subsequent years, enters the stream as groundwater discharge (Barnhart et al., 2016; Miller et al., 2021a; Brooks et al., 2021; Wolf et al., 2023). Rain contributes runoff to streamflow; however, even in areas receiving a substantial proportion of their total annual precipitation during summer in association with the North American monsoon, only a small proportion of the total precipitation makes it to the stream (Solder and Beisner, 2020; Tulley-Cordova et al., 2021) – most is evaporated from soils or transpired by plants (Milly and Dunne, 2020). Thus, lower-elevation streams, particularly later in the summer, depend heavily on groundwater discharge from higher elevations to sustain their flows (Miller et al., 2016), and the majority of streams in lower-elevation, arid areas are likely to lose water to shallow groundwater recharge (Jasechko et al., 2021).

Within this hydrologic framework, human water use and management introduces complexity via reservoirs and managed release schedules; trans- and interbasin transfers, conveyances, and surface and groundwater withdrawals; and irrigation for agricultural crop or turf grass growth. Turf irrigation in cities composes the majority of household water use in most municipalities, and agricultural irrigation can comprise up to 80 % of total statewide water use in western US states (Dieter et al., 2018). Water used for agricultural crop or turf grass growth locally intensifies water balance fluxes via increases in both water application and evapotranspiration in these select tracts of land. Depending on the method, both agriculture and turf grass irrigation can contribute to local groundwater recharge (Grafton et al., 2018), with greater recharge coming from flood irrigation compared with sprinkler or drip irrigation methods. Water for irrigation can come from either surface or groundwater withdrawals. The irrigation water source may have both direct and indirect influences on streamflows, particularly during low-flow seasons, and may, depending on conditions, contribute to streamflow increases, decreases, or delays in discharge (Essaid and Caldwell, 2017; Condon and Maxwell, 2019; Ketchum et al., 2023). However, these processes and fluxes are not currently explicitly included in the NWM.

Past NWM evaluations have leveraged stream gauge measurements (Hansen et al., 2019; Seo et al., 2021; Towler et al., 2023), and model evaluation using stream gauge measurements is included in the NWM WRF-Hydro workflow (Gochis et al., 2018). Using measured discharge to evaluate the NWM is useful because the data are publicly available at a high spatial and temporal resolution (e.g., dataset used in Towler et al., 2023). However, evaluation of streamflows with measured discharge (1) may allow modelers to get the correct total streamflow values and temporal patterns at a reach for the wrong process reasons or (2) may suggest that the model could be improved due to mismatches between measured and modeled data, but it cannot provide information on the specific process(es) or sources responsible for the errors.

Among the climatic regions covered by the NWM, model streamflow evaluation metrics perform the most poorly in lower-elevation reaches in the western US. Metrics like the Kling–Gupta efficiency (KGE) indicate pervasive mismatches between measured and modeled streamflows, while the percent bias (PBIAS) results show that simulated streamflow volumes tend to be overestimated in the west (Towler et al., 2023). Similarly, Hansen et al. (2019) found that the NWM has difficulty estimating flows during drought or low-flow years in the Colorado River basin. In the low-elevation stream reaches of the western US, disagreement between the NWM flows and observations within anthropogenically altered reaches may come from the incomplete representation of anthropogenic water sources or processes in the NWM.

In the western US, low-elevation waterways have a moderate to high potential for anthropogenic alteration (Fergus et al., 2021). Rivers and surface water supplies are managed by dams, and a large proportion of total water use is allocated to irrigating agriculture (Dieter et al., 2018). However, the NWM does not explicitly include surface water removal for agricultural irrigation nor subsurface return flows from irrigation in its streamflow computations. Likewise, the NWM represents inflow and outflow of lakes and reservoirs as passive storage and releases, with no active reservoir management. Both of these omissions may be contributors to the large errors observed in the NWM in lower-elevation areas where land use includes large amounts of along-river agriculture and streamflow is heavily managed through reservoir operations. Unfortunately, the effects of contributions of these two water sources on streamflow are difficult to identify and quantify through evaluations of streamflow records alone.

Elemental or isotope ratios in media associated with hydrologic processes (i.e., water, dissolved gases, suspended sediments, and dissolved ions) are used to track the contributions of specific water sources (e.g., groundwater and runoff) to rivers or other surface waters (Cook and Solomon, 1995; Hall et al., 2016; Gabor et al., 2017). Tracers are useful because they provide information that is otherwise impossible to disentangle from direct measurements of streamflow.

Stable water isotopes (O and H) have been used to extract hydrologic process information (Jasechko et al., 2014; Evaristo et al., 2015) and diagnose process limitations in other modeling contexts (Nusbaumer et al., 2017; Putman et al., 2019). Water comprises three commonly measured stable isotopologues: light-atom-bearing H21O16 (the most abundant) as well as heavy-oxygen-bearing (H21O18) and heavy-hydrogen-bearing (H1H2O16) isotopologues. Measurements of stable water isotopes use the ratio of the heavy to light isotopologue for each atom: R=O18∕O16 or H2∕H1 expressed in delta notation (δO18 and δH2), where $\delta =1000\cdot (\frac{R_{\text{sample}}-R_{\text{standard}}}{R_{\text{standard}}})$. Samples with higher ratios may be described as “enriched” with respect to an isotope relative to a reference, whereas those with lower ratios may be described as “depleted” with respect to an isotope and relative to a reference.

The utility of any tracer comes from its spatial and temporal variability. In the case of water isotopes as tracers, variability arises from isotopic fractionation, a physically governed “sorting” of heavy-atom-bearing water molecules (H21O18 and H1H2O16) from those bearing only light atoms (H21O16), that occurs during phase changes (i.e., evaporation, condensation, sublimation, deposition; Bowen et al., 2019). Spatial and temporal patterns of δO18 and δH2 are very similar, as evidenced by the strong correlations between δO18 and δH2 in precipitation (Craig, 1961; Putman et al., 2019) and in other waters, including those in the ground, surface, and soil (Evaristo et al., 2015; Tulley-Cordova et al., 2021).

Linear relationships between δO18 and δH2 in precipitation and in waters derived from precipitation (e.g., ground, river, lake, and soil) are the basis for the ubiquitous water line (WL) framework, in which the best fit lines of the form δH2=βδO18+I are calculated for different water types (e.g., meteoric water line, MWL; ground water line, GWL; and surface water line, SWL) and are defined either for specific points (local, e.g., local meteoric water line LMWL) or for regional or global datasets (e.g., global meteoric water line, GMWL) comprising multiple points. Slopes and intercepts of these lines have useful physical interpretations (Putman et al., 2019), particularly as they relate to the global average conditions. Global average conditions are represented by the GMWL, which has a slope of 8 and intercept of 10. Differences between δO18 and δH2, relative to an expected, global average relationship are calculated using a secondary parameter called deuterium excess (defined as d=δH2−8⋅δO18). Deuterium excess (d) is used to detect evaporation of precipitation and surface waters, evaporation under a vapor pressure gradient, or nonequilibrium condensation processes, like snow formation in mixed-phase clouds or isotopic fractionation during the melting of snow (Ala-aho et al., 2017; Putman et al., 2019; Bowen et al., 2018; Sprenger et al., 2024).

Because hydrologic processes including groundwater recharge, discharge, and precipitation runoff do not cause isotopic fractionation, we can use water fluxes from hydrologic models with estimates of the isotope ratios of those fluxes on the appropriate timescales to produce river water isotope estimates. This works well because the groundwater and runoff fluxes to summertime streamflow in the western US have distinct stable isotope ratios due to seasonal and spatial controls on precipitation isotope ratios. The signatures of groundwater inflow and snowmelt tend to have the lowest isotope ratios of the water sources in the hydrologic system and tend to be relatively temporally invariant (Bowen, 2008; Feng et al., 2009; Jasechko et al., 2014; Solder and Beisner, 2020; Tulley-Cordova et al., 2021). In contrast, summer precipitation, which contributes runoff to streams, tends to have higher isotope ratios than groundwater (Jasechko et al., 2014; Tulley-Cordova et al., 2021).

Anthropogenic modifiers of streamflow that are not included explicitly in the NWM (i.e., irrigation and reservoirs) may be expected to alter the isotopic signature of streamflow downstream of the headwaters. Agricultural irrigation can contribute both runoff to streams and recharge groundwater (Essaid and Caldwell, 2017; Gochis et al., 2018). Evaporation occurring during conveyance and application increases the isotope ratios in water recharged by irrigation and decreases d (Craig and Gordon, 1965; Yang et al., 2019). This isotopic signature is passed along to the plants (Oerter et al., 2017). Thus, irrigation-sourced recharge (runoff or ground) exhibits an evaporated isotopic signature that is distinct from naturally recharged groundwater or precipitation runoff. The effects of evaporation on the isotope ratios of the return flows are expected to be greater in arid areas with higher summer temperatures and higher vapor pressure deficits. Although lakes can be isotopically enriched with lower d (isotopically evapoconcentrated) relative to other surface waters (Bowen et al., 2018), we do not expect similar signals of evaporation-driven isotopic enrichment from reservoirs. Relative to natural lakes across the US, evaporation rates from western lakes are low relative to inflow (Brooks et al., 2014). Instead, reservoirs may alter the isotope ratios of streamflow through retention and later discharge of spring snowmelt. Thus, reservoir outflow may have lower isotope ratios and higher d than the upstream rivers during the summer months.

In this study, we compared hydrologic-model-informed estimates of long-term mean streamflow isotope ratios with stream water isotope observations across the western US. The model-informed estimate of river water isotope ratios used an isotope mass balance methodology that combined the long-term average water fluxes of the NWM and water stable isotope datasets. If the NWM constrains all water sources affecting streamflow, we expect that the differences between the isotope mass balance results and isotopic observations (observation–model differences) will be small and uniformly positive or negative throughout each basin. If we observe spatial and/or seasonal variability and structured patterns in observation–model differences within basins (i.e., patterns with elevation, stream order, or aridity), particularly with respect to the sign of the difference, we may infer that the NWM is incorrectly partitioning runoff and groundwater fluxes or is missing important water sources. We hypothesize that, if we observe spatial variability and structured patterns in our observation–model difference data, we will observe higher isotope ratios and lower d in more arid reaches, reflecting the influence of irrigation return flows, which we expect bear an isotopic signal of evaporation, on streamflow compared with higher-elevation, humid or seasonally snowy reaches with minimal anthropogenic influence.

## 2 Methods

**Figure 1.**
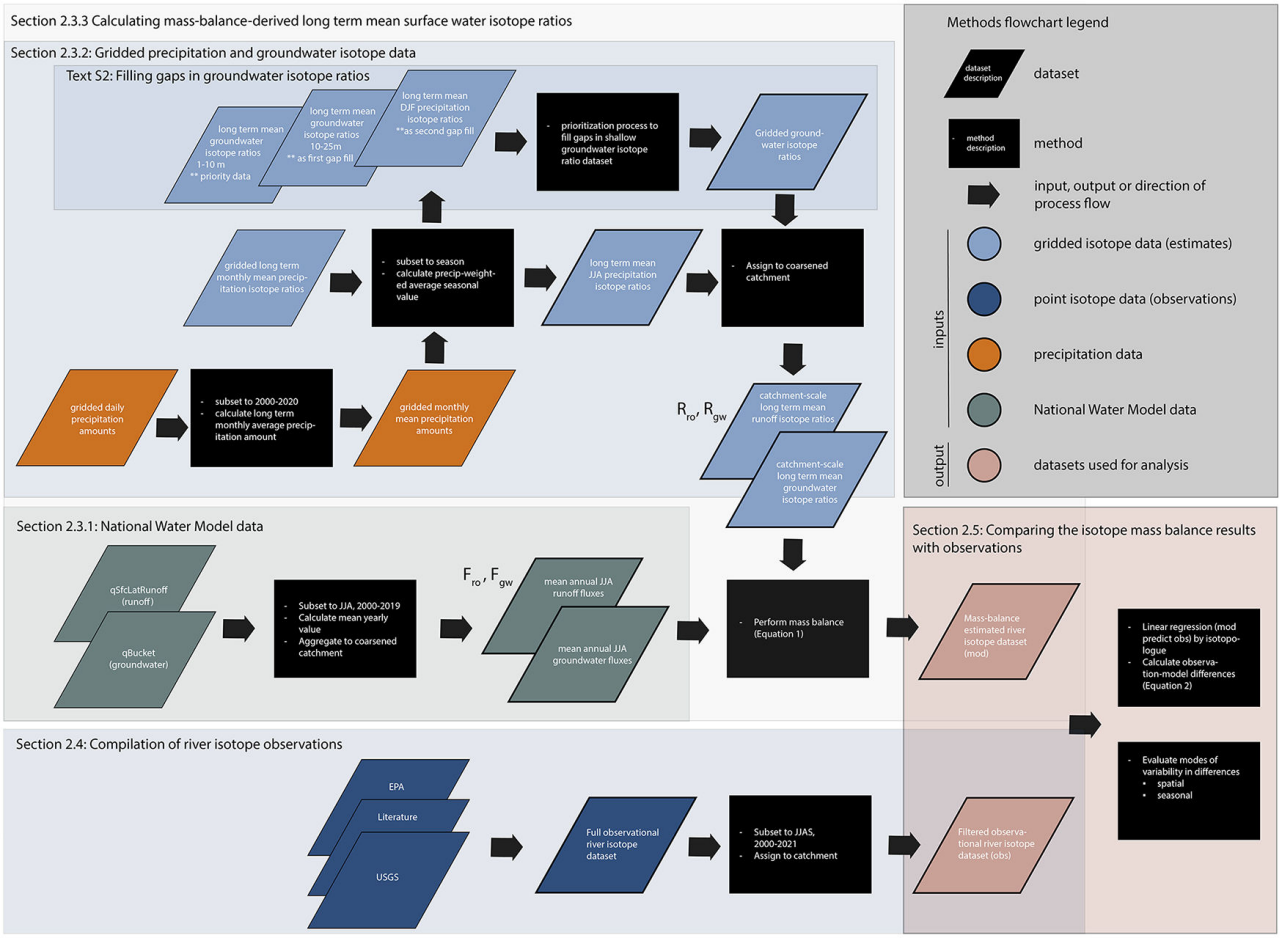
Diagram showing methods and datasets, as described in Sect. 2.3-2.5. Four data streams were used to formulate the long-term isotope mass balance estimates of river isotope ratios: gridded precipitation isotope estimates (Bowen, 2022b), gridded groundwater isotope estimates (Bowen et al., 2022), gridded precipitation data (University of East Anglia Climatic Research Unit et al., 2021), and NWM data (National Oceanographic and Atmospheric Administration, 2022). Three data categories contributed to the observational river isotope dataset: USGS (U.S. Geological Survey, 2022), EPA (U.S. Environmental Protection Agency, 2016b, 2020; Brooks, 2024), and literature datasets accessed from the WaterIsotopes database (Putman and Bowen, 2019).

This study analyzes spatial patterns in observation–model differences to evaluate missing sources of streamflow in the NWM in the western US. The “model” estimates are produced using an isotope mass balance approach, where water fluxes were supplied by NWM simulations of groundwater and surface runoff fluxes (National Oceanographic and Atmospheric Administration, 2022) and isotope ratios came from gridded groundwater and precipitation stable isotope products (Fig. 1, Sect. 2.3; Bowen, 2022b; Bowen et al., 2022). These mass balance estimates were compared to a large collection of stable river water isotope observations, and both the compiled observations and mass balance estimates are publicly available (Fig. 1, Sect. 2.4; Reddy et al., 2023). Differences between observations and modeled data were compared in an error-partitioning framework (Sect. 2.5), and we tested the hypothesis that spatial variability in observation–model differences contains a signature of agricultural water use (Sect. 2.6). A groundwater isotope ratio dataset and a well water surface elevation relative to river surface elevation dataset from Jasechko et al. (2021) were used as independent lines of evidence supporting our analysis of observation–mass balance estimate differences (Sect. 2.7).

### 2.1 Temporal domain

Our analysis was constrained to summer months (June, July, and August) between 2000 and 2019. The specific months chosen reflect those with greatest evapotranspiration, and thus consumptive water use, and correspond to the season with the largest number of spatially distributed river water isotope observations.

### 2.2 Spatial domain

We selected five basins with two-digit hydrologic unit codes (HUC2 basins) (U.S. Geological Survey, National Geospatial Technical Operations Center, 2023) in the western US to compose our study area: the Upper Colorado (14), Lower Colorado (15), Great Basin (16), Pacific Northwest (17), and California (18). All basins were characterized by rivers sustained by wintertime snowpack mediated by groundwater infiltration and discharge. All basins also included water management through impoundments and substantial water use for agriculture. In a simplified Köppen climate classification (Rubel and Kottek, 2010), the southern and central portions of the study area were characterized as arid, whereas much of the northern and mountainous portions of the study area was classified as warm temperate or seasonally snowy.

The spatial domain and streamflow routing were represented by a network of flow lines (reaches) and catchments (*n* = 15787, with one flowline for each catchment) derived from the National Hydrography Dataset Plus (NHDPlus; U.S. Geological Survey, 2019; see also Sect. S1 in the Supplement of this work for network processing details) and clipped to the spatial domain of our study. Catchments had a median size of 51 km^2^ and a mean size of 221 km^2^, and flow lines had a median length of 20 km^2^ and a mean length of 32 km^2^. All data used in this analysis were spatially joined to this network, and we retained attributes provided by NHD-Plus for analysis, including catchment area, Strahler stream order, reach length, minimum and maximum catchment elevation, and feature code, which denoted the flow line path type.

### 2.3 Using isotope mass balance to estimate long-term mean river isotope ratios

Using estimates of long-term mean groundwater and precipitation isotope ratios (Bowen et al., 2022; Bowen, 2022b), we applied an isotope mass balance to the NWM groundwater and surface runoff fluxes to streams (Fig. 1). The operational hydrologic model is based on the open-source community hydrologic model WRF-Hydro (Gochis et al., 2020a, b) and simulates and forecasts major water components (e.g., evapotranspiration, snow, soil moisture, groundwater, surface inundation, reservoirs, and streamflow) in real time across the conterminous US (CONUS), Hawaii, Puerto Rico, and the US Virgin Islands. In the NWM framework, surface and soil evaporation are wrapped into the evapotranspiration flux variable, and direct evaporation from rivers and reservoirs are not considered in the NWM surface water balance. Thus, we did not apply any additional isotopic fractionation to the groundwater and surface runoff isotopic fluxes. This approach produced an estimated long-term mean isotope ratio for river reaches in the western US. These estimates were directly comparable to river water isotope observations.

#### 2.3.1 National Water Model data

We accessed lateral surface runoff (NWM variable qSfcLatRunoff, m^3^ s^−1^) and groundwater (qBucket, m^3^ s^−1^) fluxes from the NWM v 2.1 analysis assimilation dataset (National Oceanographic and Atmospheric Administration, 2022) for our mass balance estimates (Fig. 1). The NWM runoff term (qSfcLatRunoff) only includes surface runoff and does not include subsurface runoff. Instead, subsurface runoff is routed from the bottom of the soil layer to the groundwater bucket (qBtmVertRunoff). We also accessed streamflow (streamflow, m^3^ s^−1^) fluxes as a reach-scale quantity to be included in the analyses of results. All of the NWM variables that we used are available at the NHDPlus reach scale at an hourly time step between 2000 and 2019. We divided these variables into subsets for the summer months (June, July, and August) and calculated the mean water fluxes to each reach for the summer season of each year. The interannual variability in the summer fluxes was leveraged as an estimate of the uncertainty of the long-term mean summer water fluxes.

#### 2.3.2 Gridded precipitation and groundwater isotope data

The precipitation and groundwater stable isotope ratios (δH2 and δO18) that we used to perform the isotope mass balance came from two publicly available gridded products. Both represent long-term means or climatologies and provide estimates of uncertainty.

We obtained monthly precipitation isotope ratio climatological predictions and uncertainty estimates (1 standard deviation) for both H and O from Bowen (2022b). The monthly US grids were available at 1 km and were produced with the Online Isotopes in Precipitation Calculator (OIPC) v3.2 database (Bowen, 2022a) following methods described in Bowen et al. (2005). Monthly grids have been adjusted for consistency with annual values (see version notes for OIPC2.0; Bowen, 2006). In general, isoscape accuracy depends on the spatial and temporal coverage of point datasets available to produce the isoscape. The Bowen (2022b) product is the highest-resolution gridded product available for CONUS and, in contrast to other global or regional gridded isotope products, is produced using precipitation isotope ratio data from not only the Global Network of Isotopes in Precipitation (GNIP) but also the US Network of Isotopes in Precipitation and a host of other precipitation samples collected and stored in the WaterIsotopes database (Putman and Bowen, 2019). In our input dataset, the median standard deviations of both δH2 and δO18 are about 0.12 ‰, but they may be as large as 2 ‰-3 ‰, depending on the region and isotope, based on a N-1 jackknife approach to error estimation (Bowen and Revenaugh, 2003).

We calculated the precipitation-weighted long-term mean summer (June, July, and August) and winter (December, January, and February) seasonal isotope ratio climatologies with long-term monthly mean precipitation climatologies calculated from the Climatic Research Unit (CRU) mean monthly precipitation amounts (Harris et al., 2020; University of East Anglia Climatic Research Unit et al., 2021) for the period from 2000 to 2020. The precipitation-weighted mean seasonal climatology error was calculated analytically from the time series.

The groundwater isoscapes used in this analysis were produced by Bowen et al. (2022) for seven depth intervals ranging from 1 to 1000 m. The groundwater isoscapes were not temporally resolved. The authors report errors smaller than 0.71 ‰ and 1.07 ‰ in δO18 and δH2 estimates, respectively, based on a cross-validation approach. The approach was validated using an independent dataset, and it was found that variance in the modeled groundwater predicts 92 % of the variance in the validation dataset, with no bias. The authors suggest that, as it estimates groundwater isoscapes at different depth intervals, the approach results in more accurate estimates than methods for producing bulk groundwater isoscapes.

Because this project focuses on groundwater discharge to streams, we preferentially utilized the 1–10 m depth interval. However, this layer contained some data gaps where insufficient well data were present to perform an estimate. Where available, we filled these data gaps using either other groundwater depths or mean winter precipitation (December, January, February), as described in Sect. S2. The groundwater isotope ratio data included estimates of uncertainty, which were retained for the characterization of uncertainty around the mass balance isotope ratio estimates.

The gridded precipitation and groundwater isotope datasets and their uncertainties were assimilated to the NHD-Plus spatial framework. Because the raster data grid sizes were larger than the catchment sizes, we employed a distance minimization approach using the centroid of the catchment and the centroids of the grid cells.

#### 2.3.3 Calculating mass-balance-derived long-term mean surface water isotope ratios

To estimate the long-term mean surface water isotope ratio ($R_{\text{sw},r}$) at each reach (r) in the spatial domain (Eq. 1), we accumulated the groundwater (gw) and surface runoff (ro) isotope fluxes (i.e., the isotope ratio multiplied by the water flux, R⋅F) for all reaches (i) from the headwaters downstream to the reach. The isotope ratio for surface runoff ($R_{\text{ro}}$) came from the summer mean gridded precipitation isotope ratios, whereas the isotope ratio for the groundwater flux ($R_{\text{gw}}$) came from the gridded groundwater isotope ratios (see Sect. 2.3.2). The summed isotope fluxes were divided by the summed surface runoff and groundwater fluxes.


(1) $$R_{\text{sw},r}=\frac{\sum_{i=0}^{r}R_{\text{gw},i}\cdot F_{\text{gw},i}+R_{\text{ro},i}\cdot F_{\text{ro},i}}{\sum_{i=0}^{r}F_{\text{gw},i}+F_{\text{ro},i}}$$

Our long-term mean estimates of $R_{\text{sw},r}$ are subject to uncertainty from (1) interannual variations in the mean summer volumetric contributions of groundwater and surface runoff to streamflow and (2) because the long-term mean estimates of the groundwater and precipitation isotope ratios are subject to uncertainty arising from underlying data coverage as well as interannual variability. To constrain uncertainty in our long-term mean estimates of $R_{\text{sw},r}$, we calculated 200 estimates of $R_{\text{sw}}$ per reach by taking 10 random draws from the isotope ratio distributions (assuming a normal distribution) for each of the 20 years of record. This approach uses (1) interannual variability in surface runoff and groundwater fluxes to constrain the variability in the water flux component of the calculation and (2) uncertainty in the isotope ratio estimates to constrain the uncertainty in the isotope ratio component of the calculation. Joint distributions (of either H and O or isotopes with water fluxes) were not used because information about how the isotope ratios might covary was not available from the gridded isotope datasets and no assumptions were made about how the isotopes might vary with interannual variability in climatic conditions. Similarly, no assumptions were made that the precipitation and groundwater isotope ratios covaried in time. These 200 estimates were used to calculate a long-term mean estimated isotope ratio for river water in each reach of the network and to evaluate uncertainty in our estimates.

### 2.4 Compilation of river isotope observations

The results of the mass balance calculations were compared with observations of stable water isotope ratios from rivers collected between 2000 and 2021, during the growing season months of June, July, August, and September. We included 2 additional years (2020 and 2021) as well as data from the month of September beyond the temporal constrains of the NWM model domain in our set of observations. This decision was made to maximize the number of data and the number of unique river reaches in the spatial domain that are available for analysis, and it reflects the assumption that the long-term mean river isotope ratios calculated from the mass balance approach will be insensitive to the inclusion or exclusion of a small number of additional years or an additional growing season month.

We compiled surface water stable isotope (δH2 and δO18) measurements from various sources, including the Environmental Protection Agency (EPA), the United States Geological Survey (USGS) National Water Information System (NWIS; U.S. Geological Survey, 2022), and published datasets assimilated in the WaterIsotopes database (Putman and Bowen, 2019). Not all reaches had one or more stable water isotope observations, and river reaches with multiple stable water isotope ratio observations were sometimes, but not always, from the same sampling site within the catchment.

The EPA surface water stable isotope data came from the National Rivers and Streams Assessment (NRSA; U.S. Environmental Protection Agency, 2016b, 2020; Brooks, 2024) and the National Lakes Assessment (NLA; U.S. Environmental Protection Agency, 2009, 2016a; Brooks, 2024). These data were collected once or twice per summer on a 5-year rotating basis as part of routine sampling campaigns. Over the time period of our analysis, we obtained three collections of NRSA samples (2008–2009, 2013–2014, and 2018–2019). Sites were sometimes, but not always, resampled among the campaigns. Sampling was stratified based on the Strahler stream order and by state, ensuring that all orders were sampled within each state in the assessments (U.S. Environmental Protection Agency, 2016b, 2020). This means that higher-order reaches are less frequently sampled than medium- or low-order reaches.

The USGS surface water stable isotope data for rivers were downloaded via the NWIS application programming interface (U.S. Geological Survey, 2022), and the literature data came from published and unpublished sources that are publicly available through the WaterIsotopes database (Putman and Bowen, 2019). Stable isotope collections are not part of routine measurements for the USGS; rather, these values are collected by specific USGS projects. Thus, stable isotope data collections from the USGS and literature datasets tended to be spatially and temporally clustered.

### 2.5 Comparing the isotope mass balance results with observations

The relationships of the NWM isotope mass balance (modeled) to the river isotope observations were evaluated using correlation and simple regression analyses, where the modeled isotope ratio (either δH2 or δO18) values were used to predict the observed isotope ratios. We evaluated the results with all unaveraged observations and the mean isotope ratio at river reaches with multiple observations. A Pearson correlation analysis was performed using the “corr()” function of Python’s “pandas” package (McKinney, 2010; The pandas development team, 2020). Regression analysis was performed using the ordinary least squares (OLS) function in the Python “statsmodels” package (Seabold and Perktold, 2010).

We calculated the likelihood that an observation and the model result came from the same distribution, based on the variance in the model estimate, and the variance associated with river water isotope observations (Sect. S3) using a twotailed *t* test. We report *p* values, where *p*<0.1 indicates that the isotope mass balance estimate was statistically different from the observed surface water isotope ratio for the specific element (H or O).

#### 2.5.1 Calculating observation–model differences

**Figure 2.**
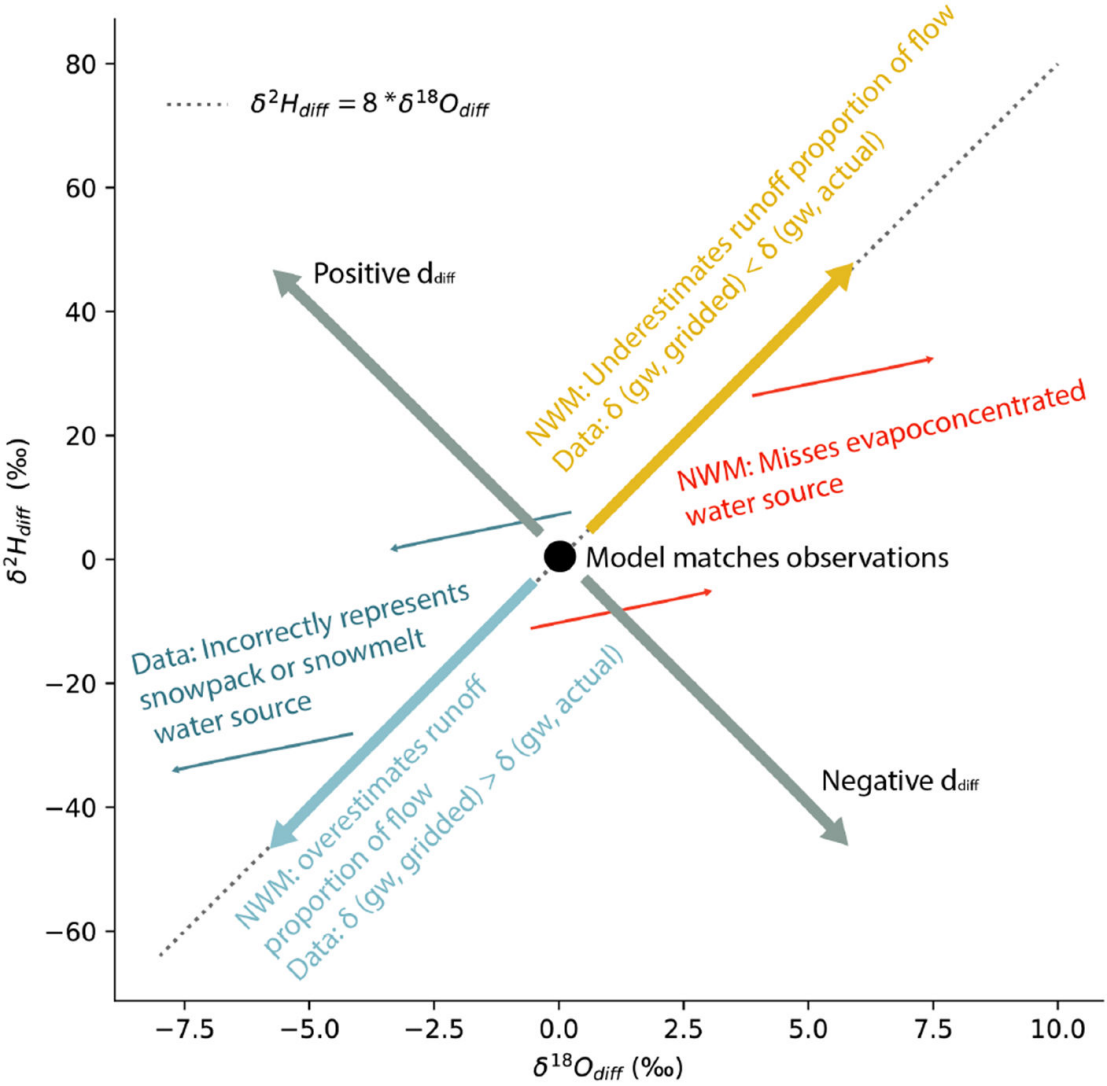
Schematic for interpretations of observation–model differences utilizing dual-isotope difference space and assumptions about the expected relationships between δOdiff18 and δHdiff2. The annotations associated with “NWM” specify the sort of hydrologic model error (i.e., water source apportionment) that could produce the observation–model comparison result if all isotope data supplied to the isotope mass balance are correct. The annotations associated with “Data” specify the sort of error in the gridded isotope datasets that could produce the observation–model result if all NWM water source contributions are assumed to be correct. The interpretations of the secondary mode of variability, captured by $d_{\text{diff}}$, depend on the model producing results that reflect equilibrium relationships between δO18 and δH2.

We calculated the observation–model (obs–mod) estimate differences in both δO18 and δH2 by subtracting the model estimate from the observation (δOdiff18=δOobs18−δOmod18;δHdiff2=δHobs2−δHmod2). Using both isotope systems, we established a framework for the interpretation of our results (Fig. 2) that utilizes movement along or deviation from the global mean δH2:δO18 ratio of 8 that is used to represent fractionation that occurs at equilibrium and defines the slope of the global meteoric water line (GMWL; Craig, 1961).

Observation–model differences may arise from either (1) incorrect model source representation (i.e., missing water sources or incorrect fluxes of established sources) or (2) errors in the isotope ratio datasets used for the isotope mass balance calculation. Thus, for positive or negative values of δOdiff18 and δHdiff2 that exhibit a δHdiff2:δOdiff18 ratio of 8, we infer either errors in the NWM with respect to the proportions of surface runoff and groundwater contributed or errors in the gridded isotope ratios (likely groundwater, due to its disproportionate contributions to streamflow). For positive or negative δOdiff18 and δHdiff2 with δHdiff2:δOdiff18 ratios different from 8, we infer that the NWM is missing uncharacterized water sources with isotope values bearing a signature of nonequilibrium fractionation. We quantify differences in the δHdiff2:δOdiff18 ratios from 8 using a metric similar to d called $d_{\text{diff}}$ (Eq. 2).


(2) $$d_{\text{diff}}=\delta^{2}H_{\text{diff}}-8\cdot \delta^{18}O_{\text{diff}}$$

We can interpret combinations of δOdiff18 and $d_{\text{diff}}$ together as well as $d_{\text{diff}}$ independently to infer the uncharacterized sources responsible for the observation–model difference. This framework is useful because the ratios of δH2 to δO18 of the isotopic inputs to the isotope mass balance tend to be close to 8 (Bowen, 2022b; Bowen et al., 2022), whereas those from the observations more often differ from 8 (U.S. Environmental Protection Agency, 2016b, 2020). This means that all nonzero $d_{\text{diff}}$ values can be used to identify omitted water sources with nonequilibrium fractionation signals and can be used to diagnose where these sources may contribute to streamflow. The conditions of this study, based on the data and approach, mean that the mass balance approach represents a null hypothesis that all processes and sources contributing to streamflow carry an isotopic signal of equilibrium fractionation (i.e., precipitation, groundwater, and routing). In other instances, where the modeled approach could reflect a combination of equilibrium and nonequilibrium processes, the interpretation of observation–model differences, particularly in terms of the $d_{\text{diff}}$ axis, may change.

### 2.6 Evaluating variability in observation–model differences

Following the spatial strength of our dataset, which relies heavily on the EPA NRSA datasets, we focused on evaluation of spatial variability in observation–model differences in our dataset. We evaluated temporal variability to (1) support findings from our analysis of spatial variability and (2) determine whether there may be spatial–temporal covariance that influences our results.

The spatial structure in the observation–model differences was evaluated graphically by comparison of δOdiff18 and $d_{\text{diff}}$ with catchment mean elevation, Strahler stream order, and Köppen climate class (Rubel and Kottek, 2010). The former two variables were retained from the NHDPlus catchment dataset (U.S. Geological Survey, 2019). The Köppen climate class was joined to the spatial framework, as described in Sect. S4.

The spatial structure in the observation–model differences was also evaluated statistically with linear mixed-effects modeling using the basin (HUC2) as a random variable with the Python statsmodels module and the “mixedlm()” function (Seabold and Perktold, 2010). Linear mixed-effects modeling with basin as the random (grouping) variable was selected for the analysis method because water in streams at low elevations is likely to be more isotopically similar to water in the basin headwaters than a nearby stream in a different basin with different water source regions. Thus, we assume the groups are likely to have different mean values reflecting their hydrologic and climatic differences. Although we also expect that the relationship of the response variable $d_{\text{diff}}$ to the explanatory variables may differ among basins, both our response and explanatory variables contain substantial scatter as well as small numbers of high-leverage points in each basin, such that a more nuanced analysis that includes temporal aspects of variability would be likely to produce misleading results.

Using the linear mixed-effects approach, we tested the statistical relationship between $d_{\text{diff}}$ and the ratio of actual evaporation to precipitation ($\frac{{\text{ET}}_{a}}{P}$; Sect. S4), catchment mean elevation (Elev), fraction of streamflow estimated to come from agricultural return flows ($F_{\text{irr}}$, Sect. S5), and a categorical variable indicating the influence of large reservoirs (Res, capacity > 6.1674 × 10^6^m^3^; Sect. S5.2). We performed statistical analysis on all streams not categorized as intermittent, ditches, or canals.

To assess how the observation–model difference may change over the growing season, in which the relative fraction of agricultural water in a waterway may increase due to low flows and increased water use, we obtained all site–year combinations in which there were at least three observations during at least 3 of the 4 months (June–September) of the growing season. We required 1 of the months be the month of June. From the June value(s) of δOdiff18 and $d_{\text{diff}}$ for a site–year combination, we subtracted the δOdiff18 and $d_{\text{diff}}$ values calculated for other months at the same site and from the same year. We evaluated the distribution of the aggregate results as well as the distributions at the HUC2 basin scale by comparing their means and inter-quantile ranges.

Interannual variability was also assessed (Sect. S6) to ensure that patterns in the other modes of variability did not arise due to either covariability in spatial and temporal patterns of sampling or the timescale difference between our isotope mass balance estimates (long-term mean) and observations (instantaneous).

### 2.7 Evaluation of independent lines of evidence supporting the signature of agricultural water use in rivers

Because it is difficult to disentangle the effects of elevation and aridity from the effects of human water use and management due to their spatial covariance, we utilized analyses of independent datasets to support the results of our statistical inference. The analyses evaluated relationships between land use or cover and groundwater isotope ratios and the fraction of well water levels that are below the nearby river level in catchments across the western US.

#### 2.7.1 Associating groundwater stable isotope observations with land use/land cover types

Estimates of the isotopic evapoconcentration of groundwater associated with different land use and land cover classes supports our inferences from observation–model differences. We made the associations between groundwater isotope ratios and land use classes at a HUC12 scale (U.S. Geological Survey, National Geospatial Technical Operations Center, 2023).

We considered five land use type categories that were aggregations of two or more National Land Cover Database (NLCD; Dewitz and U.S. Geological Survey, 2021) categories. The “desert” category was composed of the barren land (NLCD code of 31), shrub/scrub (52), and grasslands/herbaceous (71) land classes. The “forest” category was composed of evergreen, deciduous, and mixed forests (41–43). The “developed” category was composed of all the developed classes, including open (21–24). The “agriculture” category was composed of pasture/hay (81) and cultivated crops (82). The final category, “water and wetlands” comprised all other land types, including open water (11), perennial ice/snow (12), woody wetlands (90), and emergent herbaceous wetlands (95). We assigned the dominant land use/land cover category for each HUC12 using data based on the land use type with the greatest fractional coverage.

We compiled groundwater stable isotope (δO18 and δH2) measurements from the USGS NWIS (U.S. Geological Survey, 2022) and from published datasets assimilated in the WaterIsotopes database (Putman and Bowen, 2019). The groundwater isotope ratio observations were spatially joined to the hydrologic units. We did not place temporal or well depth constraints on the samples used in our analysis. Not imposing well depth constraints may contribute to scatter associated with differences in water sources recharging shallow groundwater compared with deeper confined aquifers.

#### 2.7.2 Evaluation of NWM groundwater discharge with well level fractions

The Jasechko et al. (2021) dataset compared river surface elevations with river-side well water elevations within catchments. The approach produced the fraction of wells in a catchment whose water surface levels were lower than the water surface level of the nearby river. In catchments where most well water levels are below the river water level (scores close to 1), we expect the river to lose water to shallow groundwater recharge under the right geologic conditions (e.g., permeability). In contrast, in catchments where most well water levels are above the river water level (scores close to 0), we expect groundwater discharge to streams.

We predicted the long-term mean summer NWM “qBucket” magnitude using the Jasechko et al. (2021) dataset and a simple linear regression. This approach tests the hypothesis that, if NWM accurately represents groundwater discharge to streams, the relationship of well water elevations to river surface elevation would predict the summer mean NWM groundwater discharge flux (assuming a linear relationship between the two quantities), with some scatter to account for subsurface permeability and spatial variability in groundwater discharge rates. We then evaluated the effect of agricultural irrigation in a catchment on the relationship between the NWM qBucket (binned by to the 0–20th, 20–40th, 40–60th, 60–80th, and 80–100th percentiles) and the Jasechko et al. (2021) dataset. The evaluation was split into reaches influenced by irrigation sourced from groundwater and irrigation sourced from surface water as well as reaches uninfluenced by irrigation water. Irrigation contributions and irrigation water sources were determined using the methods for estimating irrigation water use described in Sect. S5.1 and used elsewhere in our analysis.

## 3 Results and discussion

### 3.1 Evaluation of the isotope mass balance approach for estimating surface water isotope ratios

**Figure 3.**
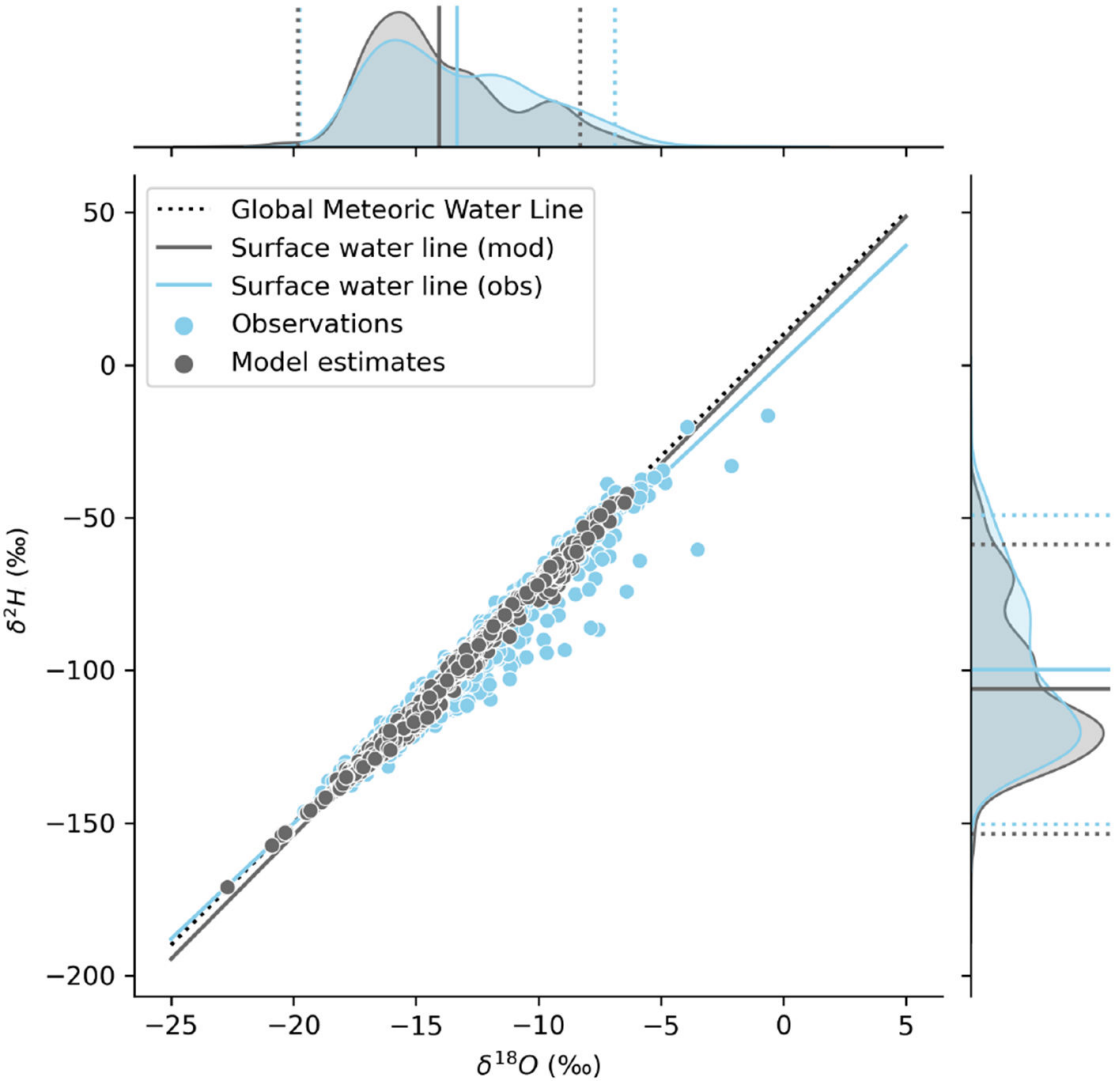
The distribution of the catchment mean observation (obs, blue) and isotope mass balance estimates (mod, gray) (*n* = 448) with the global meteoric water line (dotted) and the two datasets’ surface water lines (solid lines). See Table 1 for water line statistics. Data distributions, including the mean and 2 standard deviations of each data type (dotted lines), are shown in the plot margins. Observations plotting below the GMWL indicate evaporation, while those plotting above the GMWL may indicate mixed-phase cloud processes or other nonequilibrium condensation processes (Putman et al., 2019).

Our analysis evaluated 4503 stream stable isotope observations in 877 unique river reaches across the western US relative to NWM-driven isotope-mass-balance-derived estimates (hereafter referred to as “modeled”) of the river isotope ratios. Of these, 448 reaches had more than one observation (often all at the same sampling site in the catchment, although sometimes at multiple sites; Fig. S1) and up to 571 observations in a catchment (Figs. S1, S2). On average, across all data, the observations were significantly greater than the modeled values, by 0.537 ± 0.033 *‰* and 4.81 ± 0.222‰ for δO18 and δH2, respectively (Fig. 3). For δO18, we observed a standard deviation of 3.16 ‰ for the observed data and 2.96 ‰ for the modeled data (for all data averaged by catchment). For δH2, we observed a sample standard deviation of 25.4 ‰ for the observed data and 24.4 ‰ for the modeled data (for all data averaged by catchment; Fig. 3).

We calculated surface water lines (SWLs) for both the modeled and observed results using all available data (Fig. 3). The observations yielded an SWL with a slope of 7.570 (±0.023) and intercept of 1.2301 (±0.320), which was significantly different from the GMWL slope of 8 and intercept of 10 but was within the range of local MWL (LMWL) slopes for western North America (6.5–8) (Putman et al., 2019), as reported in Table 1. The model results yielded a SWL with a slope of 8.12 (±0.010) and an intercept of 8.06 (±0.14), which was more similar to, although still statistically different from, the GMWL and differed from LMWLs for the region (Table 1). Comparison of the observation and modeled data distributions and water lines reveals evidence of evaporation of surface waters in the observations but not in the isotope mass balance results (Fig. 3). This is because the primary source of streamflow in the modeling framework, high-elevation groundwater discharge, does not bear an evapoconcentrated isotopic signature in our input dataset, and lower-elevation water sources (groundwater or surface runoff) that could bear an isotopic signature of evaporation, depending on the region, are considered by the model to be minor contributors to streamflow over the timescale integrated by our study.

Despite the differences in the data distributions, the modeled isotope ratios and observed isotope ratios were well correlated (Table 2, Figs. S4-S7), with correlation coefficients between 0.761 and 0.866, depending on the isotopologue and whether individual observations or catchment means were considered. These correlations translated to statistically significant simple linear regressions where the modeled isotope ratios were used to explain the observed isotope ratios (Table 2). Depending on the isotopologue and whether individual observations or means were considered, the models explained between ~ 58 ‰ and 75 % of the variance in the observations. The model explained more variance for δH2 than for δO18 and explained more variance for catchment mean values relative to individual observations. For all regressions, the slopes ranged from 0.879 to 0.937, with catchment mean slopes tending to be lower than slopes calculated from all observations. Intercepts for all regressions were close to, but less than, zero, with lower intercepts associated with regressions calculated from catchment mean values, relative to regressions calculated from all observations. The statistically significant slopes of less than 1 and statistically significant intercepts arise in all observation–model comparison regressions because the observations tended to exhibit higher isotope ratios than the model estimated at the lower end of the isotopic distribution (Figs. S4-S7). Many of the catchments characterized by this pattern were in arid regions. The greater variance explained by the regressions using catchment means relative to the individual observations suggests that using temporally varying inputs rather than calculating a long-term mean river isotope ratio may further improve observation–model comparisons.

### 3.2 Observation–model differences

Of 4503 observations, 1763 δO18 and 3306 δH2 observations were significantly different from the long-term mean isotope mass balance NWM estimate at *p*<0.1. Of these, 1756 observations indicated significant differences for both δO18 and δH2. This corresponded to a median absolute difference of 2.2 ‰ for δO18 and 9.7 ‰ for δH2. For both, a larger proportion of the distribution indicated positive significant differences, and those differences tended to be greater in absolute magnitude than the negative significant differences.

**Figure 4.**
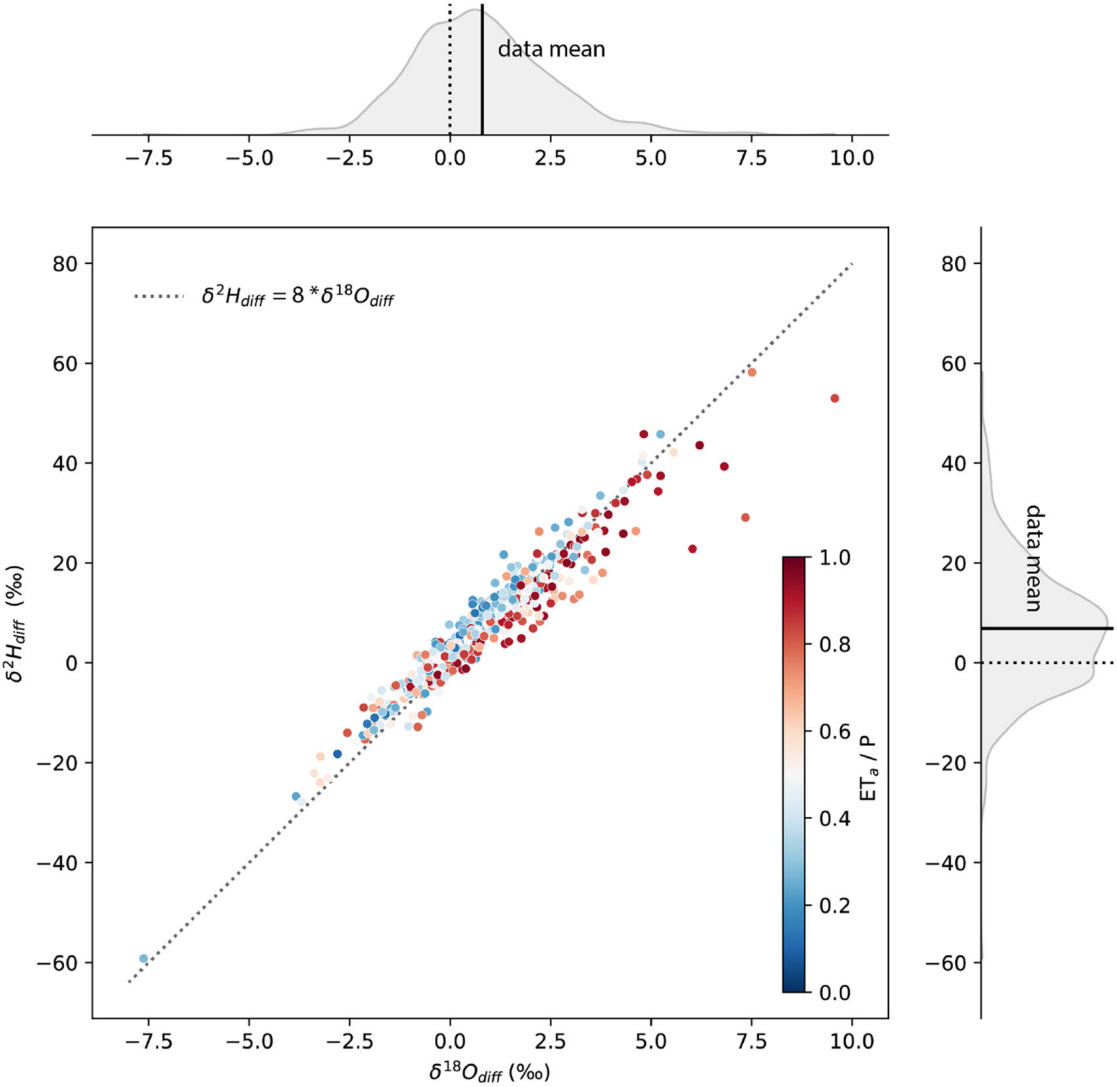
The relationship of observation–isotope mass balance estimation differences for δO18 and δH2. Interpretations of the scatterplot follow the framework indicated in Fig. 2. The catchment mean value is plotted, and only sites with at least two observations are shown (*n* = 448). The equilibrium line with a slope of 8 is plotted for context (dotted line), and data are color-coded by their site’s ratio of actual evaporation to precipitation. Data distributions are shown for both δOdiff18 and δHdiff2 in the margins, while the mean differences are indicated as a solid line. No difference (0) is marked with a dotted line for reference.

We used an observation–model difference interpretation framework (Fig. 2) to gain process information that can be used to improve our understanding of terrestrial water balance and process inclusion in the NWM. The observation–model differences in δO18 and δH2 were correlated (Fig. 4) and yielded similar results for analyses performed with all data compared with means of reaches with multiple observations (Table 2). Simple linear regressions, where variance in δOdiff18 explained variance in δHdiff2, with all data and catchment mean data both explained about 92 % of the variance, were significant, and exhibited slopes of less than 8 (Table 2), suggesting the presence of errors arising from NWM omission of water sources that bear signatures of nonequilibrium processes.

In our dataset, model estimates do not deviate much from the GMWL, and they deviate less than the observations (Fig. 3). The model estimates reflect an assumption that water sources contributing to streamflow were subject only to equilibrium fractionation, whereas observations indicate contributions of waters influenced by nonequilibrium processes. This information is quantified using $d_{\text{diff}}$ (Fig. 2). Positive values of δOdiff18 tended to be associated with negative values of $d_{\text{diff}}$ (Fig. S8). The shape of the relationship between the two quantities is nonlinear, with a stronger relationship between δOdiff18 and $d_{\text{diff}}$ among data from arid reaches compared with humid reaches.

The relationship between δOdiff18 and $d_{\text{diff}}$ as well as the results of our regression (Table 2) and surface water line analyses (Table 1) indicate that the modeling approach for estimating long-term isotope ratios of rivers returns results that are similar to (but on average lower and exhibit less variability than) observations. The strongest signal in our data is that of evaporation, evidenced by combinations of positive δOdiff18 and negative $d_{\text{diff}}$ in arid regions. We also observe evidence of nonequilibrium condensation processes in reaches characterized by negative δOdiff18 and positive $d_{\text{diff}}$.

We suggest that patterns in δOdiff18 and $d_{\text{diff}}$ contain useful model diagnostic information that can be useful for improving the NWM and our understanding of the terrestrial water balance. However, the observational dataset is composed of a nonuniform compilation that contains spatial, seasonal, and interannual modes of variability. Due to the underlying sample collection approaches, the strength of our dataset is evaluating spatial variability, so we focus our analysis on that mode to gain information about missing water sources that may influence the model. We support our findings using the temporal evolution of observation–model differences through the growing season. Based on an analysis of the interannual variability (Sect. S6) we suggest that the spatiotemporal structure of our data is sufficiently robust and evenly distributed with respect to interannual variability to support the analysis. Additional sources of variability are discussed in Sect. S7.

### 3.3 Spatial distribution of observation–model differences

If the NWM fully constrained all relevant water sources, we expect to observe similar values of δOdiff18 and $d_{\text{diff}}$ throughout each basin, irrespective of the location of the observation in the basin. This is because the majority of water discharged to streams in these basins comes from higher-elevation water source areas, and (based on the assumptions of the NWM framework) little addition or modification of river waters is expected downstream of headwater catchments. Thus, we expect that the observation–model differences calculated in headwater areas would propagate to lower-elevation areas in the absence of additions from unconstrained water sources and/or river water modifications from unconstrained processes.

**Figure 5.**
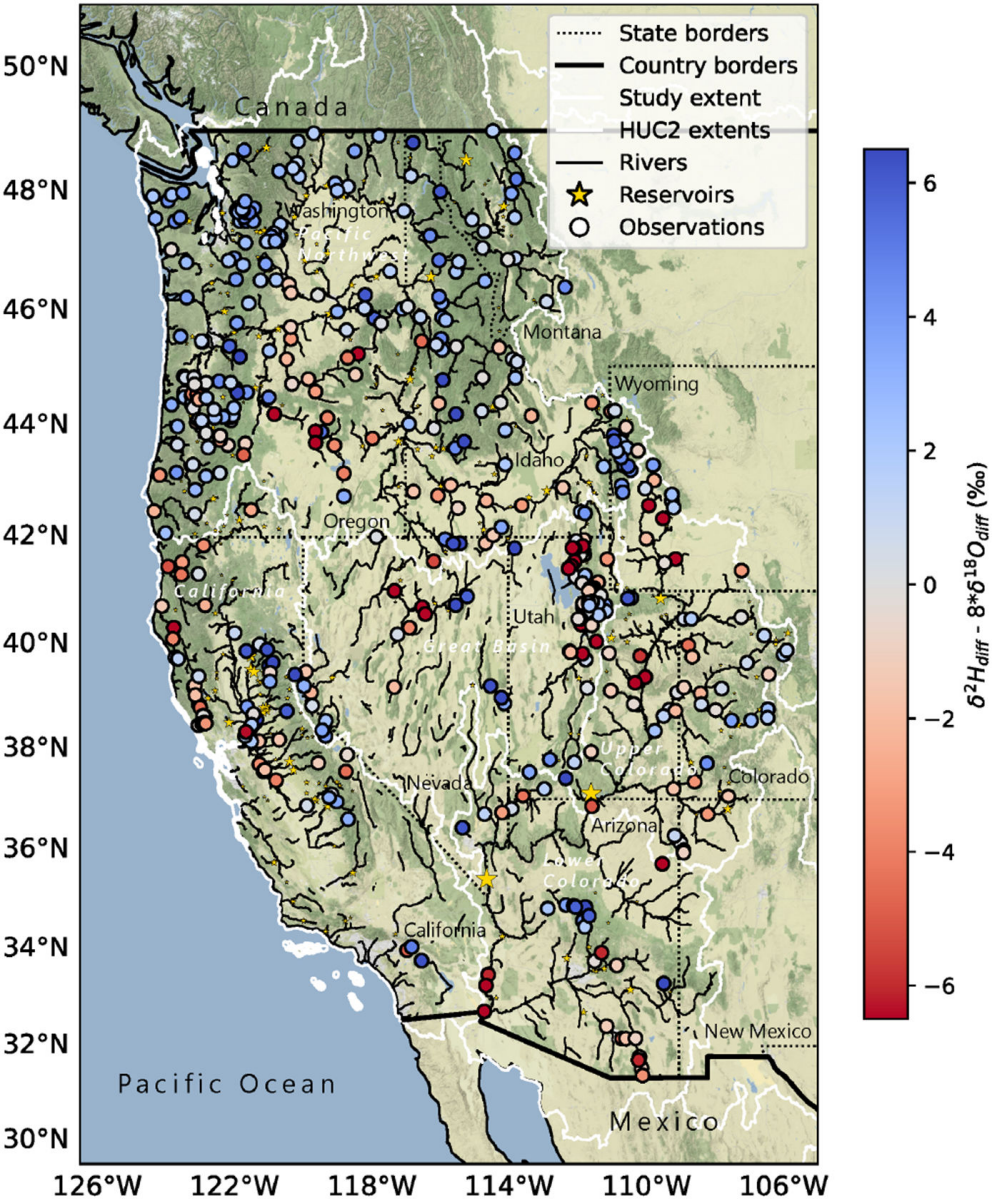
The spatial distribution of mean catchment $d_{\text{diff}}$ (δHdiff2−8⋅δOdiff18) in reaches with more than one observation (*n* = 448). Reservoirs are marked by yellow stars, with the star size proportional to the reservoir capacity. Redder symbols correspond to waters with stronger evaporation signals than expected based on the model estimate. Map data are from © OpenStreetMap contributors (2023), distributed under the Open Data Commons Open Database License (ODbL) v1.0, and accessed through Stamen Open Source Tools (https://stamen.com/open-source/, last access: 2 August 2023). HUC2 basins come from the Watershed Boundary Dataset (U.S. Geological Survey, National Geospatial Technical Operations Center, 2023), and rivers are modified from the NHD-Plus streamline network (U.S. Geological Survey, 2019).

**Figure 6.**
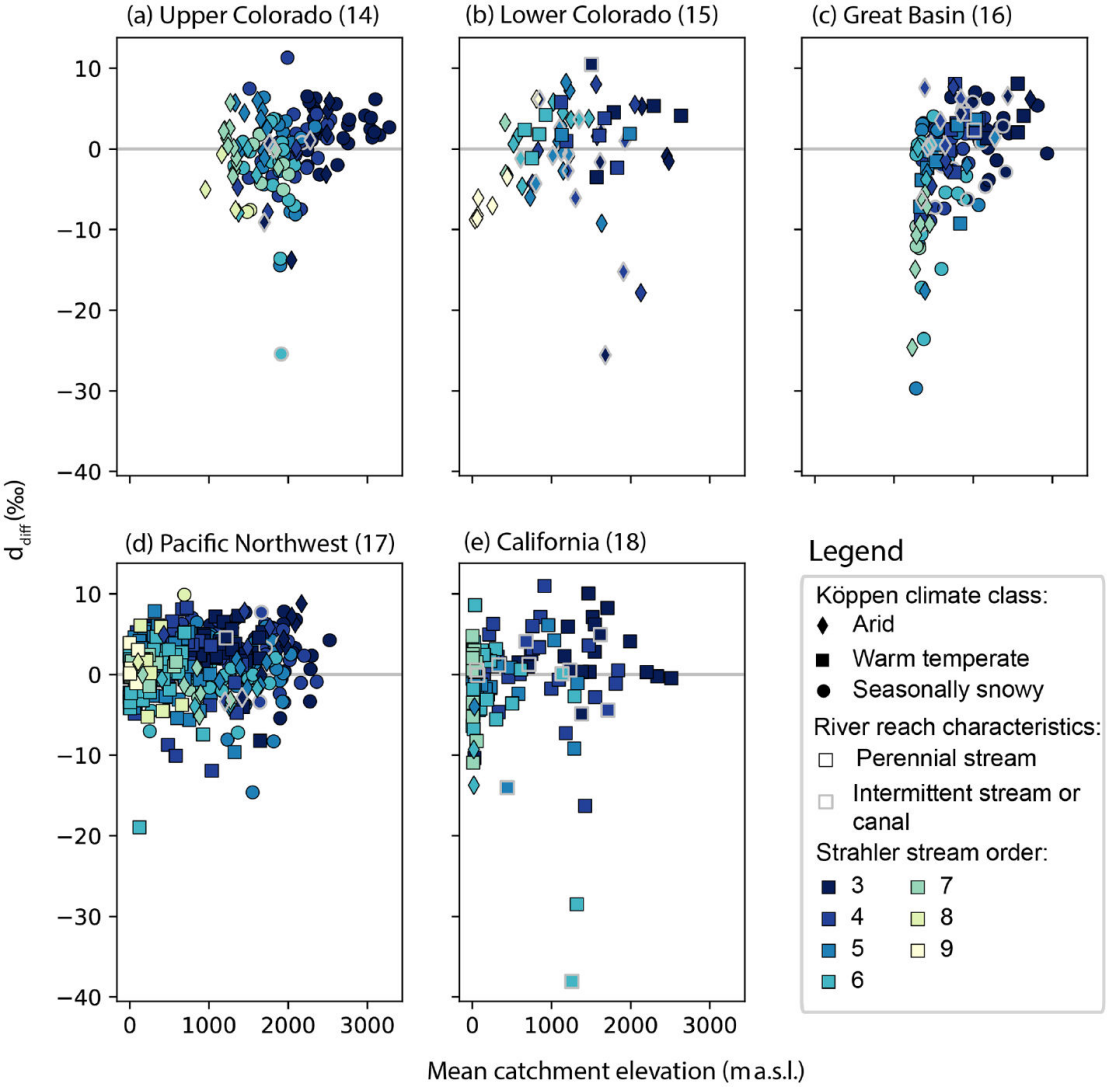
Relationship of elevation, Strahler stream order, Köppen climate classification (Rubel and Kottek, 2010), and stream persistence with $d_{\text{diff}}$ in each basin. We observe higher $d_{\text{diff}}$ in perennial, lower-order streams at middle and higher elevations in each basin. Lower $d_{\text{diff}}$ is associated with higher-order streams at lower elevations in each basin. This effect was greater in catchments classified as arid or seasonally snowy compared with those classified as warm temperate. This pattern was generally true in each basin, irrespective of the absolute elevation or stream order, suggesting the importance of accumulated effects within a basin on $d_{\text{diff}}$.

Instead, we observed spatial variability (Figs. 5, S9), where smaller-magnitude δOdiff18 values occurred in the highest-elevation, lowest-stream-order, and least-arid reaches, whereas larger-magnitude, often positive δOdiff18 values occurred in lower-elevation, arid or intermittent-flow reaches (Fig. S10). $d_{\text{diff}}$ tended to exhibit higher values in higher-elevation, lower-stream-order reaches, and lower values in lower-elevation, more-arid, higher-stream-order reaches (Fig. 6). We observed a greater range in the absolute magnitudes of δOdiff18 and $d_{\text{diff}}$ in higher-order, lower-elevation reaches (Figs. 6, S10). Notably, the pattern was similar across basins, suggesting the importance of within-basin processes in determining δOdiff18 and $d_{\text{diff}}$, as opposed to absolute relationships of δOdiff18 and $d_{\text{diff}}$ to elevation, stream order, or climate classification.

The spatial pattern in $d_{\text{diff}}$ (Fig. 5) was similar to the pattern observed for the KGE and other metric evaluations of the NWM (Towler et al., 2023). Areas with negative $d_{\text{diff}}$ tended to correspond to areas with poor NWM performance (Towler et al., 2023). However, the isotopic evaluation of NWM and the Towler et al. (2023) datasets could not be directly compared due to there being only a small number of reaches with both isotope observations and daily discharge measurements.

The spatial structure of our results was statistically well explained by the ratio of actual evaporation to precipitation ($\frac{{\text{ET}}_{a}}{P}$) in a linear mixed-effects model with basin as the grouping variable (Table 3). Variability among basins explained 16.2 % of the variance in $d_{\text{diff}}$, while the fixed effect of aridity explained 13.9 % of the variability in the dataset. The regression slope associated with the fixed effects of aridity was negative (−7.87 ± 0.78) and significant (*p*<0.01), indicating that sites with higher aridity indices tended to exhibit a more negative $d_{\text{diff}}$. This regression was stronger than a linear mixed-effects model with elevation predicting $d_{\text{diff}}$, where the fixed effects of elevation explained 4.7 % of the variability in $d_{\text{diff}}$.

Analysis of the spatial variability in our results suggests that (1) higher-elevation, lower-stream-order, perennial, warm temperate or seasonally snowy reaches had small δOdiff18 and positive $d_{\text{diff}}$ values and (2) lower-elevation, higher-stream-order, arid and sometimes intermittent stream reaches had larger and more positive δOdiff18 values and more negative $d_{\text{diff}}$ values. The first point suggests errors associated with the challenges of providing input values at appropriate temporal resolutions, including representing direct snowmelt contributions to streamflow (Sprenger et al., 2024), whereas the second point suggests that the model is missing critical evapoconcentrated water sources in more arid, lower-elevation areas of each basin.

#### 3.3.1 Observation–model differences in headwater reaches reflect groundwater isotope ratio estimates

We observe δOdiff18 and $d_{\text{diff}}$ values that are statistically different from zero in higher-elevation, low-stream-order, low-aridity, temperate or seasonally snowy reaches in our dataset (Figs. 6, S10). These differences tend to be smaller than the full dataset mean $d_{\text{diff}}$ and δOdiff18. In most of these reaches, we also observe positive δOdiff18 values (Figs. 5, 6).

The presence of both negative and positive values of $d_{\text{diff}}$ likely reflect interannual variability in the isotope ratios of actual groundwater and snowmelt discharged to rivers in high-elevation headwater areas. Although groundwater’s contribution to streams is conceptualized to be constant in magnitude and isotope ratio in this study, the isotope ratios of both groundwater and snowmelt fluxes vary spatially and interannually. The groundwater flux magnitudes vary inter-annually based on variations in snowpack magnitudes, antecedent hydrologic conditions (Brooks et al., 2021; Wolf et al., 2023), and hydrogeologic (Gentile et al., 2023) controls, including hydrologic residence times. Snowpack isotope ratios vary in response to climate patterns and local conditions (Anderson et al., 2016) and the imprint of snowmelt on river isotope ratios depends on the melt timing and contributing elevations (Sprenger et al., 2024). The observed variability in d does not exhibit a uniform tendency towards positive or negative values. This suggests that the mean groundwater isotope ratios used in this study are reasonably representative of the long-term mean estimates of the isotope ratios of water contributed at high-elevation water source areas by groundwater and snowmelt fluxes, although improvements may be made by using a temporally varying approach, where estimates of groundwater and snowmelt isotope ratios vary with month and year. However, the systematic positive d result cannot be explained by the timescale of the isotope input.

**Figure 7.**
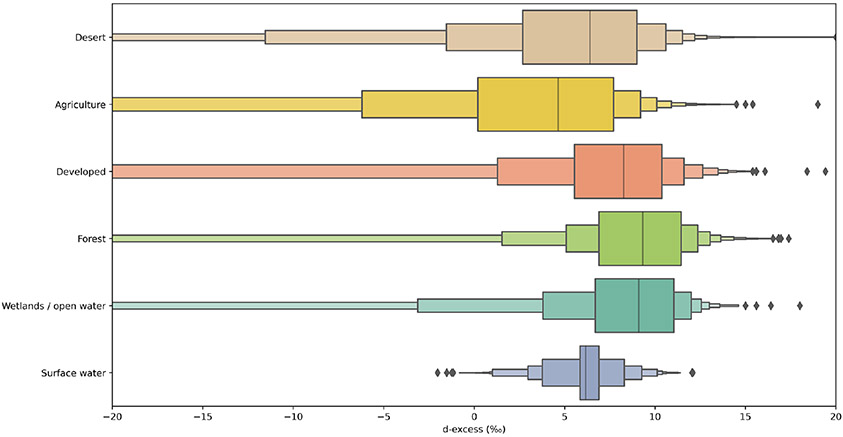
Distributions of groundwater d observations grouped by their NLCD land type (Dewitz and U.S. Geological Survey, 2021). The data are displayed as letter-value plots (Heike Hofmann and Kafadar, 2017), where the central line is the data median, the innermost box contains 50 % of the data, and the remaining boxes each contain 50 % of the remaining data (and thus a diminishing proportion of the total data, i.e., 25 %, 12.5 %, 6.25 %, etc). The black diamonds represent outliers. The plot contains between 85 % and 95 % of the data available for each land type and, thus, reasonably represents the distribution of d associated with groundwater from each land use type, even though samples with very low d are not shown. The desert land class includes barren land (often playa or dried lake bed), shrub/scrub, and grasslands/herbaceous vegetation. The agricultural land class includes pasture/hay and cultivated crops. The developed land class includes developed land of any intensity. Forest includes evergreen, deciduous, and mixed forest. The wetlands/open-water land class category includes any type of wetland as well as open water. The distribution of our 4303 river samples is also shown for context.

Higher-δO18 streamflow relative to weighted-mean precipitation values have been documented in other studies (Nickolas et al., 2017). This may be because higher d is associated with lower precipitation d that falls during the cold season in midlatitude regions, particularly in areas near open water (Putman et al., 2019; Corcoran et al., 2019; Aemisegger and Sjolte, 2018). Secondarily, high d in rivers relative to precipitation or groundwater may be attributed to fractionation occurring during melt. The snowmelt process has been demonstrated to begin with the preferential melt of water molecules bearing lighter isotopologues and to exhibit higher d earlier in the melt season (Ala-aho et al., 2017; Beria et al., 2018; Carroll et al., 2022). Further, a recent study suggested that this signal may be used to identify the elevation of snowmelt contributing to streamflow during the melt season (Sprenger et al., 2024). The higher δOdiff18 of the snow and initial meltwater may be passed along to the rivers via direct surface runoff to streams or through shallow groundwater recharge and rapid discharge to streams (see the relatively higher upper bound on $d_{\text{diff}}$ values for forested land use types in Fig. 7).

#### 3.3.2 Isotopic signals of evaporation at low elevations suggest the contribution of irrigation return flows to streamflow

Greater spatiotemporal variability in both δOdiff18 and $d_{\text{diff}}$ in lower-elevation, higher-stream-order, arid reaches suggests the importance of various spatially and temporally heterogeneous processes and water sources that may alter streamflow isotope ratios relative to upstream values. Positive values of d and negative values of $d_{\text{diff}}$ in more arid regions of each basin suggest that evaporated waters comprise a nontrivial fraction of streamflow in these areas (Figs. 5, 6, S9, S10), especially in the later part of the growing season (Fig. 9) when streams depend more heavily on groundwater fluxes. We observed isotopic evidence of contributions of evaporated waters to rivers in all basins (Fig. 6), although this was most apparent in Lower Colorado River basin, lower-elevation regions of the Upper Colorado River basin, California’s Central Valley, near Great Salt Lake in the Great Basin, and throughout the Snake River Plain (Figs. 5, S9).

The isotope ratios and $d_{\text{diff}}$ values that we observe in low-elevation, high-stream-order, arid reaches are similar to those that we would expect to observe in highly evaporative contexts, like within lakes (Bowen et al., 2018), intermittent-flow rivers, or downstream of wetlands. However, the majority of rivers in our study are perennial, and most are not characterized by substantial wetlands. The evapoconcentration in our dataset is unlikely to arise from river or reservoir evaporation, as both evaporation of reservoirs and evaporation to inflow ratios in the region tend to be low, especially for deep artificial reservoirs (Brooks et al., 2014; Friedrich et al., 2018). Instead, isotopic evidence of evapoconcentration occurs in waterways likely to be affected by anthropogenic hydrologic alteration (Fergus et al., 2021) and characterized by larger fractions of “young water” (Jasechko et al., 2014; Burt et al., 2023; Xia et al., 2023).

**Figure 8.**
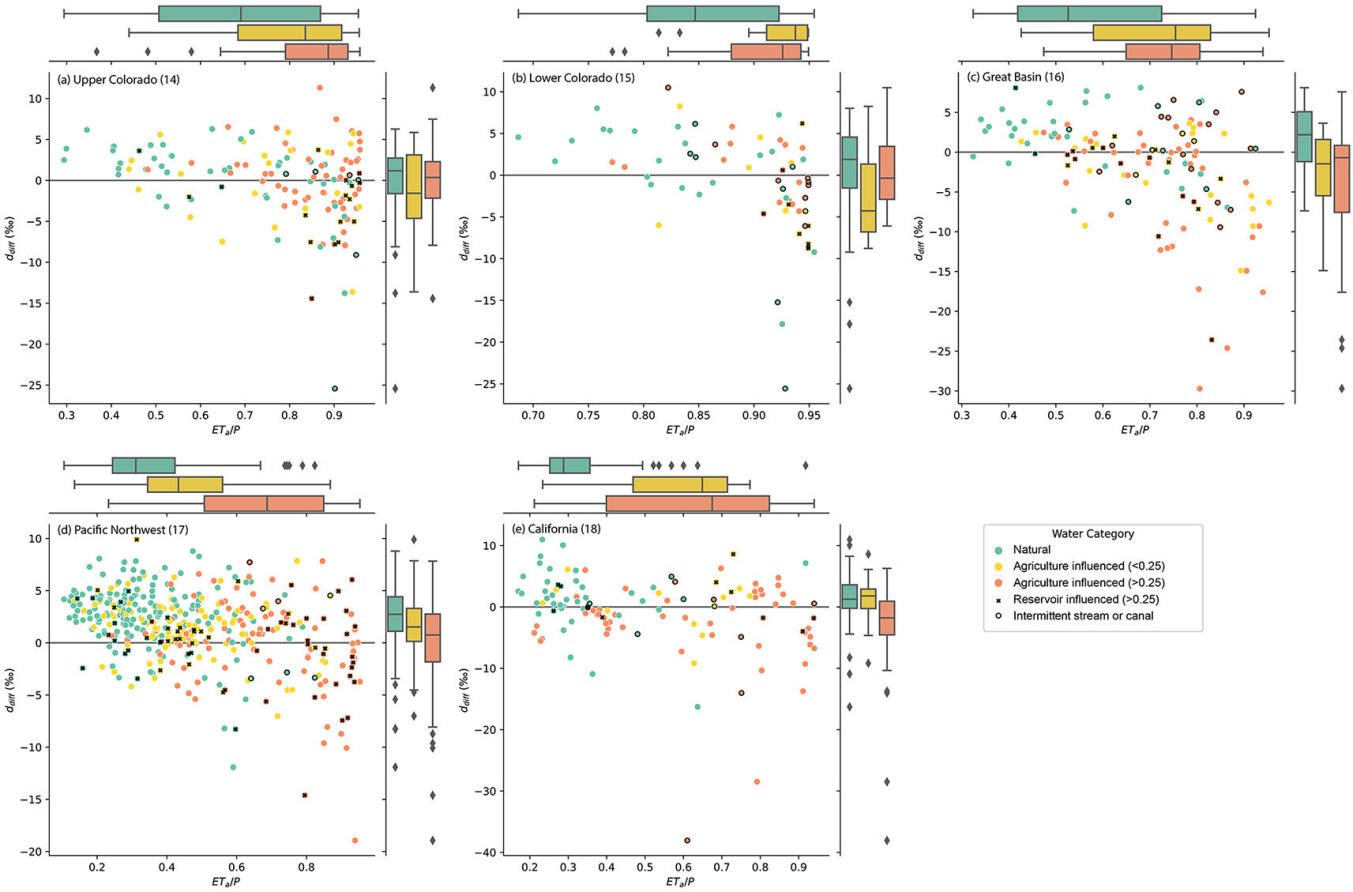
Relationship of ET_a_, a measure of aridity, with $d_{\text{diff}}$, by water use category and basin. Natural waters are not estimated to be influenced by agricultural irrigation. The fractions of agricultural irrigation contributing to streamflow are estimated using water use data and land cover data and do not account for losses to evapotranspiration. We identified reaches affected by large reservoirs (> 6.1674 × 10^6^ m^3^) and reaches categorized as intermittent or as canals or ditches with additional symbology.

We tested the hypothesis that the spatial pattern of isotopically inferred evaporation could arise from contributions of irrigation return flows to streams and reservoir releases. Within each basin, on average, $d_{\text{diff}}$ was most negative, indicating isotopic evidence of evaporation, at sites with the highest proportion of total inflows attributed to agricultural return flows, and it was highest at sites with no apparent contributions of agricultural return flows (Fig. 8). Reservoir influence was associated with low $d_{\text{diff}}$ more often in regions where dams are used for water management and water supply (e.g., Upper Colorado, Lower Colorado, Great Basin, and California) and was associated with high $d_{\text{diff}}$ in the Pacific Northwest, where dams are more often used for hydropower. Intermittent streams and canals in arid regions were sometimes associated with low $d_{\text{diff}}$ as well, even when no water was contributed by agricultural irrigation.

We demonstrated the relationships of agricultural and reservoir influence on $d_{\text{diff}}$ statistically in a linear mixed-effects model (Table 3). The fraction of streamflow estimated to come from agricultural irrigation return flows and a categorical variable delineating reservoir influence together explained 8.0 % of the variance in $d_{\text{diff}}$, with the whole model (including random group effects) explaining 14.3 % of the variance in the dataset. Both explanatory variables were significant (*p*<0.01) and, as expected, exhibited negative slopes, indicating that greater agriculture and reservoir influences tended to produce lower $d_{\text{diff}}$ values, suggestive of evaporative effects. When we included the ratio of actual evaporation to precipitation with these explanatory variables, all three are significant (*p*<0.01) and explain 15.2 % of the variance through fixed effects as well as 23.0 % of the variance overall (fixed and random effects). Among the linear mixed-effects models tested, it exhibited the highest log-likelihood value, explained the greatest amount of variance using fixed effects, and reduced the amount of variance attributed to random within-basin effects.

While this statistical model performance is not substantially better at explaining variance in $d_{\text{diff}}$ than the model that uses aridity alone, the findings do suggest that both agricultural activity and reservoirs influence the isotope ratios of streamflows across the western US. The low variance explained by these models is expected, due to the difficulty involved with estimating the true long-term mean agricultural return flux with the spatiotemporal resolution of the available data, the confounding influences of season and year on the response variable, the potential for isotopically heterogeneous reservoir effects, the covariance of both irrigation return flows and the presence of reservoirs with aridity and elevation, and the spatially variable effect of irrigation on streamflows (Ketchum et al., 2023). The statistical linkage between irrigation water use and the isotopic response would likely be improved by taking a temporally variable approach to (1) estimating river isotope ratios and (2) the contribution of irrigation water in the river., which may be doable with improvement to both precipitation isotope datasets and higher-spatiotemporal-resolution irrigation water use datasets (e.g., Haynes et al., 2023).

### 3.4 Further evidence supporting irrigation contributions to streamflow

We have statistically quantified isotopic evidence for irrigation contributions to streamflow. However, the statistical model performance is not substantially better at explaining variance in $d_{\text{diff}}$ than the model that uses aridity alone. To further investigate our findings, we include analyses of additional lines of evidence. We evaluate signals embedded in seasonal patterns in our dataset (as well as those of other studies), spatial variability in groundwater isotope ratios, and evaluation of the NWM with a well level relative to river level dataset.

#### 3.4.1 Seasonal patterns in observation–model differences

**Figure 9.**
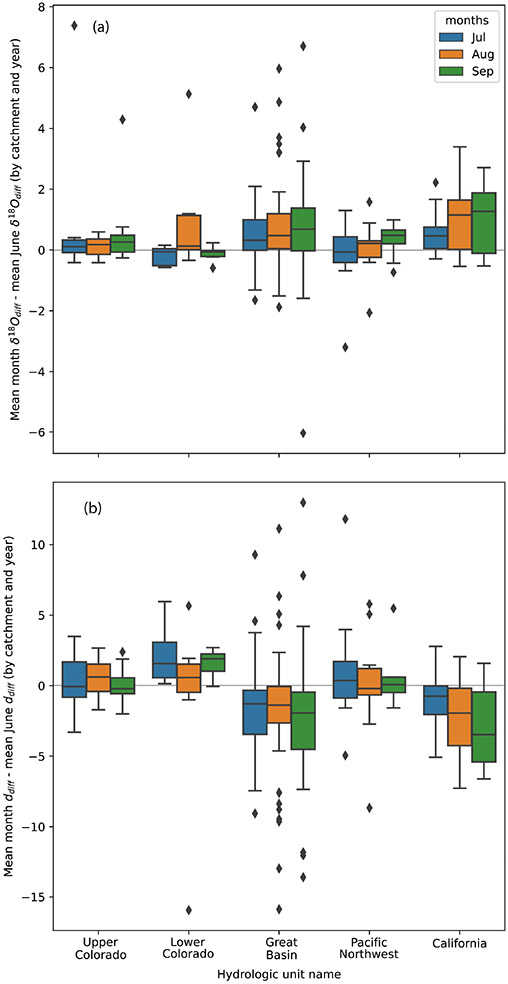
Evaluation of seasonal variability in observation–model comparisons. Data include all reaches and years with collections in the month of June as well as 2 of the 3 other months of the summer season. **(a)** The distribution (represented by box plots) of month-specific differences from June δOdiff18 by basin. **(b)** The distribution (represented by box plots) of month-specific differences from June $d_{\text{diff}}$. The box plots show the median (line), the 25th and 75th percentiles (the box), points that lie within 1.5 inter-quantile ranges (IQRs) of the lower and upper quartile (the extent of the whiskers), and observations that fall outside this range (displayed as diamonds).

There are systematic patterns in δOdiff18 and $d_{\text{diff}}$ when examined across the growing season that support our spatial assessment of the contributions of irrigation to streamflow. For example, δOdiff18 tends to be greater during the latter months of the growing season relative to the mean δOdiff18 value for the month of June for that site and year (Fig. 9a) in most basins and months. The pattern is especially evident in the Great Basin and California. Likewise, $d_{\text{diff}}$ is lower in July, August, and September, relative to June (Fig. 9b), in the Great Basin and California. The contrast between basins with both increased δOdiff18 and decreased $d_{\text{diff}}$ (Great Basin and California) and those with only increased δOdiff18 and little change in $d_{\text{diff}}$ (Upper and Lower Colorado and Pacific Northwest) suggests that two different mechanisms may drive isotopic change during the growing season.

In California and the Great Basin, which are characterized by δOdiff18 increases and $d_{\text{diff}}$ decreases over the growing season relative to June, we suggest increased contributions of evaporated waters to rivers later in the growing season. In California, this may reflect the water use and irrigation return flows contributing to streamflow in the Central Valley.

In the Upper and Lower Colorado and Pacific Northwest, where we observe small δOdiff18 increases and little $d_{\text{diff}}$ change relative to June, we suggest sustained dependence on groundwater discharge from high elevations to streamflow during the growing season (Miller et al., 2016; McGill et al., 2021; Windler et al., 2021). In downstream sections of the Upper Colorado and the Lower Colorado, where rivers are characterized by discharges from large reservoirs, the seasonal invariance may reflect that the primary “water source” regions for these reaches are reservoirs, which retain snowmelt from early in the season and discharge it later in the season.

#### 3.4.2 Literature and other datasets

Numerous prior studies have investigated the influence of irrigation on streamflow. Estimates suggest that, depending on the irrigation type, as much as 50 % of applied water may recharge groundwater and/or arrive at surface waters through shallow groundwater infiltration and subsequent discharge to streams (Grafton et al., 2018). Likewise, irrigation has been demonstrated to increase streamflows during low-flow periods (Fillo et al., 2021; Essaid and Caldwell, 2017) if the applied water comes from surface water diversions.

Local contributions of groundwater to streams from irrigation-based recharge are supported by the d values of groundwater in agricultural regions. Groundwater from regions influenced by agricultural irrigation exhibited lower mean d relative to deserts, including dried terminal lake and playa areas; developed areas, which may include turf grass irrigation; forested regions; wetlands or open waters; and surface waters (Fig. 7). Based on the isotope ratios of groundwater in irrigated areas and prior isotopic inference (Windler et al., 2021), we hypothesize that inclusion of irrigation-recharged groundwater discharge as a source of water to streams in the NWM would decrease the difference between modeled and observed isotope ratios in our dataset.

The isotopic inference that irrigation return flows are an important missing process in the NWM is supported by an independent statistical comparison of the NWM groundwater discharge with the Jasechko et al. (2021) dataset and the agricultural water use data. The Jasechko et al. (2021) data are the fraction of well water levels that lie below the proximal river water level in a catchment and provide some estimate of hydraulic head and direction of groundwater–surface water exchange. When the fraction is high, the river (under correct permeability conditions) would be expected to lose water to groundwater, whereas the river would be expected to gain water from groundwater discharge when the fraction is low.

We hypothesize that, if the NWM accurately represents groundwater discharge to streams, the Jasechko et al. (2021) well water level comparison to stream water level dataset should be able to predict the summer mean NWM ground-water discharge flux with a large proportion of variance explained. However, the Jasechko et al. (2021) data weakly (*R*^2^ = 0.028, *p*<0.01) predict the NWM groundwater discharge rates in a simple linear regression. The regression relationship between the variables is negative, as expected, where river reaches with a greater proportion of their well water levels above proximal river water levels correspond to reaches with greater groundwater discharge fluxes (Fig. S11). Although the regression is significant, it has almost no predictive capacity, contrary to expectations.

The weakness of the statistical relationship between the Jasechko et al. (2021) dataset and the NWM groundwater discharge flux may be related to shallow aquifers, which are not considered by NWM, and/or agricultural irrigation and the water source (surface or groundwater) used for that irrigation (Fig. S12). We did not assess the potential for NWM groundwater discharge to reflect the presence of shallow aquifers. However, we observe that the influence of irrigation on groundwater levels is nonstationary, depending on both the groundwater discharge magnitude and the source of irrigation water. For this reason, the relationship is difficult to assess statistically. In river reaches where the NWM indicates little groundwater discharge (0th to 20th percentile qBucket), irrigation sourced from surface water is associated with a smaller fraction of well water levels below river level (smaller y value in Fig. S12) than those without irrigation. Conversely, in river reaches with substantial ground-water discharge (80th to 100th percentile qBucket), agricultural irrigation with water from either surface or groundwater tends to be associated with a larger fraction of well water levels below river level (larger y value in Fig. S12) compared with reaches without any agricultural irrigation. Based on these patterns, we suggest that irrigation from surface water in dry areas appears to contribute to groundwater recharge, whereas irrigation appears to contribute to decreased water table elevations in wet areas. At all groundwater discharge percentiles, surface water irrigation contributes to higher water tables, whereas irrigation from groundwater contributes to lower water tables.

Some part of this signal is regional. Reaches from more arid basins compose a greater proportion of the lower-percentile qBucket reaches, whereas reaches from humid or seasonally snowy basins compose a greater proportion of the higher-percentile qBucket reaches. However, when evaluated by basin, the relationships are similar. This finding is consistent with modeling studies: lower stream discharge when irrigation water comes from groundwater and greater stream discharge when irrigation water comes from surface water (Essaid and Caldwell, 2017). Our analysis suggests that agricultural irrigation is likely to influence groundwater levels and groundwater discharge at the landscape scale and produces gaining streams and contributes to streamflow in otherwise arid, losing reaches of rivers.

### 3.5 Implications of including irrigation return flows into NWM calculations

Our evaluation of the NWM-driven isotope mass balance calculations suggest that the NWM accuracy would be improved by including agricultural return flows in the water sources sustaining streamflow in the NWM. In effect, agricultural return flows are simply groundwater fluxes to streams that occur at lower elevations than the majority of the groundwater discharge sustaining streams. Based on the magnitudes of $d_{\text{diff}}$, these lower-elevation groundwater fluxes can sometimes be large. Because the NWM is calibrated to actual streamflows that contain these return flows, these fluxes are currently being misallocated in the model. Inaccuracies in any of the model terms or fluxes influence the model’s capacity to project accurate streamflows, particularly under nonstationary hydrologic conditions. Thus, accurate model water source inclusion, particularly at low elevations where water use and availability is most critical, has implications for the model’s utility to stakeholders, including water managers and users.

Under current conditions, agricultural return flows may be critical for sustaining streamflow late in the growing season (August or September) or during drought periods. Sustained streamflow in certain reaches is critical for (1) water access for surface water diversions and (2) water availability for species’ use. For example, the survival of protected fish species requires that waterways meet thresholds of water quality, temperature, and depth (Dibble et al., 2020). Water managers make decisions about water allocations and reservoir releases in part to meet these habitat needs (Bruckerhoff et al., 2022). Agricultural return flows have the capacity to help sustain streamflow (Fillo et al., 2021), although with potentially negative effects on water quality, through agriculture-associated salinization (Miller et al., 2017; Thorslund et al., 2021; Miller et al., 2024; Putman et al., 2024), increased concentrations of nitrate (Lin et al., 2021) and other nutrients (Stets et al., 2020), contributions of pesticide and fertilizers, or alterations to water temperature profiles. These contributions of agricultural waters contribute to sustaining flow but threaten water availability. Thus, inclusion of groundwater return flows from irrigation to rivers in the western US supports improved assessments of water availability both through improved modeling of streamflows and enhanced ability to model water quality.

Explicit inclusion of irrigation return flows will assist the NWM in better projecting streamflows during periods of hydrologic nonstationarity, as are likely to characterize the hydroclimatic elements of climate change. Nonstationary processes include hydrologic changes arising from the ongoing megadrought in the southwestern US (Williams et al., 2022), associated changes in water use for irrigation (Ketchum et al., 2023), intense precipitation events (like monsoons or major storm events) that are observed to be increasing in intensity with climate change (Pfahl et al., 2017; Demaria et al., 2019), and projected changes to future snowpack depth and melt timing (Siirila-Woodburn et al., 2021; Hammond et al., 2023). The ongoing aridification of the southwestern US is characterized by increased evapotranspiration (Milly and Dunne, 2020) and changes to groundwater recharge and discharge associated with decreases in snowpack and changes to snowpack melt patterns (Hammond et al., 2023). Understanding the groundwater flux contributions of areas with shallow water tables to streamflow during major precipitation events will help better characterize areas at risk for flooding and inform appropriate water management strategies.

## 4 Conclusions

The isotope mass balance evaluation of the NWM revealed similarities between the isotope mass balance estimated isotope ratios (modeled) and observed isotope ratios. The mass balance approach represented as much as 75 % of the variance in the observations, depending on the water isotopologue evaluated. This suggests that, on average, the NWM correctly represents the relative proportions of groundwater and surface runoff fluxes sustaining streamflow during the summer and that the gridded isotope datasets are appropriate for the analysis.

The observation–model differences exhibited a spatial and seasonal structure, suggesting that the NWM is missing important additional water sources that contribute to streamflow. Specifically, the observation–model differences that plot above the equilibrium line (Fig. 2) suggest the importance of direct contributions of snowmelt to streamflow in humid areas. Those that plot below the equilibrium line suggest the importance of groundwater sources characterized by evaporation in arid areas. We tested the hypothesis that agricultural irrigation return flows are the missing evaporated water source in arid regions and found them to be a significant predictor of observation–model differences. Future work may benefit from taking a temporally varying approach to the estimation of streamflows and agricultural contributions to streams, as the difference in timescale between the observations and models is a source of uncertainty. Nonetheless, our finding is supported by multiple lines of evidence, including the seasonality of observation–model differences, the relationship of land use to isotopic signals (d) of evaporation in groundwaters, a comparison of NWM groundwater discharge with an independent assessment of the potential for groundwater discharge, and isotopic and modeling study conclusions from the literature.

Our findings suggest that the NWM accuracy would be improved by including agricultural irrigation fluxes into the NWM water sources. Agricultural irrigation-recharged groundwater functions as a lower-elevation baseflow flux, and this flux is likely to be critical for sustaining streamflow during drought periods or late in the growing season. Inclusion of this specific source into groundwater fluxes would improve the ability to meet water manager and water user NWM data needs. Specifically, water managers use predictions of reach-specific flows at lower elevations during summer precipitation events and monsoons to assess flood risk or to inform dam releases (if dam releases are incorporated into the NWM) in order to assess the volume of water required to achieve specific management goals, like fish species preservation or dam water level maintenance for hydropower production. Likewise, the explicit inclusion of irrigation return flows in NWM calculations will assist in accurately predicting and projecting streamflows in heavily managed sections of river in the event of changing irrigation practices, increased evapotranspiration, or water supply reductions and fallowing of agricultural fields, which would change or halt irrigation groundwater fluxes. Finally, our findings have implications for areas at risk of diminished water availability due to issues of quality, arising from the entrainment of fertilizer and pesticides and as well as the dissolution and delivery of salts.

## Supplementary Material

Supplement1

## Figures and Tables

**Table 1.** Surface water line slopes and intercepts (δH2=δO18+I) compared to the global meteoric water line and published precipitation water line ranges (LMWLs) from different climate classifications in North America (data from Putman et al., 2019). Because all regressions are highly significant, no *p* values are shown. The slopes (β) and intercepts (I) with their standard error (SE) as well as the variance explained and regional slope minimum (min), maximum (max), and average (avg) values are presented.

Surface water lines	β (±SE)	I (±SE)	*R* ^2^
Model derived	8.12 (±0.010)	8.06 (±0.14)	99.3 %
Observations	7.57 (±0.02)	1.23 (±0.32)	96.1 %
Meteoric water lines	$\beta_{\min}$, $\beta_{\max}(\beta_{\text{avg}})$	$I_{\min}$, $I_{\max}(I_{\text{avg}})$	
Global meteoric water line	8	10	
Arid and temperate dry summer LMWLs	6.56, 8.02 (7.57)	−10.5, 9.85 (3.02)	
Temperate humid and continental LMWLs	7.34, 7.64 (7.49)	−3.82, 3.31 (0.62)	

**Table 2.** Correlation and regression results for observation–model comparisons. Regressions were performed on all data (*n* = 4503) as well as on the mean values in a subset of the reaches with more than one observation (*n* = 448). The number of observations (*n*), the correlation coefficient, slopes (β) and intercepts (I) with their standard error (SE), and the variance explained (*R*^2^) are presented.

Statistical model	*n*	Correlation coefficient	β (±SE)	I (±SE)	*R* ^2^
δOobs18~δOmod18+I	4503	0.761	0.917 (±0.012)*	−0.645 (±0.168)*	57.9%
δOobs,avg18~δOmod,avg18+I	448	0.820	0.879 (±0.029)*	−0.891 (±0.414)*	67.3%
δHobs2~δHmod2+I	4503	0.819	0.937 (±0.010)*	−1.90 (±1.06)*	67.1 %
δHobs,avg2~δHmod,avg2+I	448	0.866	0.905 (±0.025)*	−3.10 (±2.66)	75.1 %
δHdiff2~δOdiff18+I	4503	0.959	6.54 (±0.029)*	1.30 (±0.065)*	91.9%
δHavg,diff2~δOavg,diff18+I	448	0.958	6.70 (±0.094)*	1.46(±0.190)*	91.9%

An asterisk (*) indicates that the coefficient is significant at *p*<0.1.

**Table 3.** Results of linear mixed-effects models with 764 observations and 5 groups. The minimum and maximum group sizes were 48 and 387, respectively. Results from regressions with the elevation (Elev), evapotranspiration divided by precipitation (ET / *P*), intercept (I), fraction of river water estimated from irrigation ($F_{\text{irr}}$), and Boolean variable indicating reservoir influence (Res) are shown. The models do not include any samples from reaches characterized as an intermittent stream or canal or where the NWM indicates that the maximum streamflow is 0 m^3^ s^−1^. Random effects apply only to the intercepts. An asterisk indicates that a regression coefficient (β for slope and *I* for intercept) is statistically significant at *p*<0.01. The conditional *R*^2^ (Cond. *R*^2^) value, which gives the total model variance explained, is reported alongside the fixed *R*^2^ (Fixed *R*^2^), which gives the variance explained by fixed effects (i.e., explanatory variables), and the log likelihood, which can be used to evaluate the relative performance of different models.

Statistical model	β (±SE)	*I* (±SE)	HUC2 (group)	Cond. *R*^2^	Fixed *R*^2^	Log likelihood
$d_{\text{diff}}\sim \text{Elev}+I$	Elev: 0.001 (0.00)*	−1.93 (1.01)	4.33	20.9 %	4.4%	−2254
$d_{\text{diff}}\sim \frac{\text{ET}}{P}+I$	$\frac{\text{ET}}{P}:-7.85(0.77)$*	4.86 (1.08)*	4.37	30.2%	13.9%	−2209
$d_{\text{diff}}\sim F_{\text{irr}}+\text{Res}+I$	$F_{\text{irr}}:-3.49(0.48)$* Res: −1.75 (0.45)*	0.95 (0.59)	1.43	14.3%	8.0%	−2224
$d_{\text{diff}}\sim \frac{\text{ET}}{P}+F_{\text{irr}}+I$	$\frac{\text{ET}}{P}:-6.50(0.88)$* F_irr_: −1.60 (0.54)*	4.39 (0.83)*	1.941	22.8%	14.8%	−2204
$d_{\text{diff}}\sim \frac{\text{ET}}{P}+F_{\text{irr}}+\text{Res}+I$	$\frac{\text{ET}}{P}:-6.08(0.88)$* F_irr_: −1.67 (0.54)* Res: −1.22 (0.44)*	4.32 (0.82)*	1.861	23.0%	15.2%	−2200

## Data Availability

The data are publicly available from https://doi.org/10.5066/P9NOD5ES (Reddy et al., 2023).
