# Supplementary material for: Isotopic evaluation of the National Water Model reveals missing agricultural irrigation contributions to streamflow across the western United States
Source: Hydrol Earth Syst Sci. Author manuscript; Available in PMC 2025 Jul 4. (PMC11457154; doi:10.5194/hess-28-2895-2024)
Supplement: Supplement1 [file NIHMS2007883-supplement-Supplement1.pdf]

Supplement of Hydrol. Earth Syst. Sci., 28, 2895–2918, 2024
https://doi.org/10.5194/hess-28-2895-2024-supplement
© Author(s) 2024. CC BY 4.0 License.

Hydrology and Earth System Sciences
Open Access

Supplement of

Isotopic evaluation of the National Water Model reveals missing agricultural irrigation contributions to streamflow across the western United States

Annie L. Putman et al.

Correspondence to: Annie L. Putman (aputman@usgs.gov)

The copyright of individual parts of the supplement might differ from the article licence.

Supplemental text

Sect S1: Coarsening NHDplus

Because of the high resolution of the NHDPlus dataset relative to the spatial resolution of our surface water sample dataset and gridded isotope datasets, as well as the time requirements for performing network operations on large stream networks, we performed a coarsening operation on the NHDPlus catchment and flowline datasets. We pruned the stream network to include only reaches of Strahler stream order 4 or higher and removed secondary flowpaths at divergences. We then aggregated reaches and catchments that no longer contained branches. All reaches on stream orders of 3 and above were aggregated to coarsened catchments, and the closest order 3 reach was retained for routing. We applied the aggregation to the catchment areas (sum), reach lengths (sum), minimum (min) and maximum (max) elevations, and long term mean annual discharge (max) and included these as attributes of our coarsened network. We dropped any stream network that did not contain at least one catchment with an isotope observation. The original network, particularly in flat arid areas, contains flowline networks that are internally draining (do not reach a network with a major outlet), and did not contain a surface water isotope observation. These reaches were not included in the analysis.

Sect S2: Filling gaps in the gridded groundwater isotope ratios

We filled data gaps in the 1-10m depth groundwater isotope layer with data from the 10-25m depth interval (Bowen et al., 2022). However, both depth intervals contained data gaps in similar places (Bowen et al., 2022, see Figure 3), so some gaps remained. These gaps occurred in higher elevation, mountainous areas, as well as unpopulated, lower elevation areas, where groundwater data (i.e., levels, isotope ratios, etc) are less frequently measured, due to difficulty sampling, lack of available wells, or large depths to groundwater. In mountainous areas in the western US, groundwater is recharged through melt of wintertime snow. Thus, for the gaps in the gridded groundwater dataset in the shallowest two layers, we used the precipitation climatology-weighted average winter (December, January, February) precipitation isotope ratios as shallow groundwater isotope ratio estimates. These values may differ from the actual groundwater, considering complications with snowmelt fractionation and timing of groundwater recharge during the melt season.

Sect S3: Estimating the variability of surface water isotope ratios

Because most catchments did not have enough observations to estimate their own variance for the t-test for significance of difference between observations and modeled isotope ratios, we estimated the magnitude of both within-season and interannual variability in surface water isotope ratios, assuming a normal distribution for river isotope ratios. To evaluate the expected seasonal and interannual variability at our sites, we selected a subset of original resolution NHDPlus catchments with at least 15 observations from at least 4 different years (Figure S3). The threshold of 4 years follows evaluations of data requirements for calculating local meteoric water lines (LMWLs), which are sensitive to interannual variability. For LMWLs, 4 years was sufficient to characterize the majority of the long-term variability in precipitation (Putman et al., 2019). Because of the large contributions of groundwater to surface water, which is a less

isotopically variable water source than precipitation, interannual variability in rivers is typically much lower than for precipitation, but higher than for groundwater (e.g., Kirchner, 2016). This is most true for headwater streams, which tend to be characterized by a small fraction of young water, and less true for lower elevation streams, which may be characterized by a higher fraction of young water (Jasechko et al., 2014).

We evaluated the standard deviation of the suite of observations for the subset of catchments, and evaluated possible controls on variability, including stream order, mean annual streamflow, region, and elevation, though found no clear relationships among variability and any of the variables in our dataset. Thus, we selected the median standard deviation of our subset of sites to serve as the standard deviation at all reaches (Figure S3). Although this is unlikely to represent the true variability at all sites, it is a conservative approach to representing the interannual variability at surface water sites.

Sect S4: Assigning the Köppen climate classification and calculating the aridity metric

The Köppen climate classification (Rubel and Kottek, 2010) was assigned to each coarsened catchment by intersecting the catchments with the Köppen climate classification raster center points using a 'within' criteria for a spatial join. The climate class data were simplified to three climate types following the simplification applied in Putman et al., (2019): Subtropical arid or seasonally hot and dry regions (Köppen classes B and Cs) were termed 'Arid'. Humid temperate (Köppen class Cf) were termed 'Warm temperate' and seasonally snow dominated regions (all Köppen subclasses within D) were termed 'Seasonally snowy'.

To provide climatic context to our analysis, we obtained daily 4 km gridded precipitation (P) and actual evapotranspiration (ET_a) data from GridMet (Abatzoglou, 2013) for the years 2000-2020. The mean annual values for each quantity were computed, and an aridity metric was calculated as ET_a/P. High values of the aridity ET_a/P mean that nearly all precipitation is lost as evapotranspiration over the course of the year (typical of arid regions), whereas low values mean that total evapotranspiration is low relative to precipitation (typical of more humid regions).

Sect S5: Estimation of river water fractions

We estimated the fraction of river flow composed of three different water sources: groundwater, runoff, and agricultural return flows using NWM variables, water use (Hutson et al., 2004; Kenny et al., 2009; Maupin et al., 2014; Dieter, C.A., et al., 2018) and land use datasets (Dewitz and US Geological Survey, 2021). We also used reservoir influence as a categorical variable in our model to assess the effects of reservoir management on observed isotope ratios.

S5.1 Calculating agricultural water use at catchment scale

We used water-use data to evaluate and contextualize observation-model isotopic differences in our dataset. Water-use data were available at county scale from the 2000, 2005, 2010, and 2015 National Water Use Census' (Hutson et al., 2004; Kenny et al., 2009; Maupin et al., 2014; Dieter, C.A., et al., 2018). We used the irrigation water categories `Irrigation, total withdrawals, fresh' ($R_{irr,\ county}$) and `Irrigation, acres irrigated, total' ($A_{irr,\ county}$) in our analysis.

To convert the water-use information from county-scale withdrawal rates ($R_{irr, county}$) to the total water applied at the coarsened catchment scale ($W_{irr, catch}$, Equation S1), we first calculated the water withdrawn for irrigation per irrigated acre per day for each county. We assumed that all agricultural areas in a county used water at the same rate, and that the rate of water application did not change throughout the season, and all water that was withdrawn was applied.

$$W_{irr,catch} = A_{irr,catch} * \left(\frac{R_{irr,county}}{A_{irr,county}}\right) \quad \text{(Equation S1)}$$

To convert the county-based estimate of water use to the coarsened catchment scale, we mapped catchments to the counties they resided in using the catchment centroid and a spatial join method in python's `Geopandas' package (Jordahl et al., 2020). Because of this, catchments were mapped to a single county, even if they might span multiple counties. We then multiplied the total county-average fresh water withdrawal per irrigated acre by the total number of acres of land in a catchment categorized as `pasture/hay' and `cultivated crops' by the 2019 National Land Cover Database ($A_{irr,catch}$, Dewitz and US Geological Survey, 2021).

S5.2 Estimating accumulated water fractions comprising river flow

To calculate the estimated water fractions of the three water types (groundwater (*gr*), runoff (*ro*), and agricultural return flows (*irr*)) at each reach (*r*), we iteratively accounted for water inputs, starting at the stream network headwaters (*i=0*) and moving downstream to the reach (Equation S2). The iteration order was established using the python anytree package function 'PostOrderIter' as applied to a stream network tree composed of the coarsened NHDplus reaches. For each reach, we added the incremental water (*W*) delivered to the stream for each water type (*type*) to the total water added to the stream from all upstream reaches. To calculate the fraction of each water type that composed streamflow at the reach ($F_{r, type}$), we normalized to the total water `added' to the reach from all water types throughout the upstream network.

$$F_{i,type} = \frac{\sum_{i=0}^{r} W_{i,type}}{\sum_{i=0}^{r} W_{i,irr}+W_{i,gr}+W_{i,ro}} \quad \text{(Equation S2)}$$

For agricultural irrigation return flows, we assumed that all water applied for irrigation eventually makes its way back to the nearest waterway. That is, all water estimated to be applied for agricultural irrigation in a reach ($W_{irr, catch}$, Equation S1) was counted as contributing to river flows. This simplifying assumption is unlikely to be correct, as some irrigation water in arid regions is likely lost during conveyance to the point of use, as well as to evaporation and crop transpiration (i.e., consumptive use). It is estimated that only as much as 50% of the water applied for irrigation may make it to recharge or runoff, and that percentage depends on the irrigation type (e.g., flood, sprinkler, drip; Grafton et al., 2018) and climatic controls on evapotranspiration. A use of any constant would produce the same results. Furthermore, we expect that the isotopic evolution of the irrigation water due to evaporation during and after application will be largest in arid regions where lots of water must be applied for plant growth. Conversely, cooler, wetter areas that apply less water may also lose less water to evaporation

and exhibit a smaller isotopic evaporation signal. As a result, we expect that the isotopic fingerprint of agricultural return flows on river isotope ratios will be greater in places where more irrigation water is applied, even if much of the water does not make it back to the river. Thus, by simplifying our calculation, we can draw a more direct mechanistic relationship between irrigation practices and the isotopic response and avoid complicating estimates of actual losses, recharge and actual return flows.

We estimated the influence of reservoirs on observation-model differences by assigning a categorical variable denoting whether a site was or was not 'reservoir-affected'. A site was categorized as being 'reservoir-affected' when at least a quarter of the water in the stream was categorized as being from a reservoir. We calculated these water fractions in a second calculation using a similar method to our approach for estimating in-river fractions of the other water types (Equation S2). The difference for this approach is that when a catchment was the site of a medium or large reservoir (reservoir storage > 50,000 acre-feet, as identified and quantified by the National Inventory of Dams (Army Corps of Engineers, n.d.), we assigned all water type fractions (groundwater, runoff, and agricultural irrigation) a value of zero, and set the total reservoir water fraction to 1. Likewise, the total accumulated water from all water types were summed, and the total reservoir values was set to this number and all other water type amounts were set to 0. This operation reflects the assumption that when water spends time in a large reservoir it mixes with water delivered at different times and evaporates, so isotope ratios of river water downstream of a reservoir may be distinct from those upstream of a reservoir. This assumption may be truer for reservoirs used for agricultural or municipal uses and flood control (i.e., Lake Powell, Flaming Gorge), and may be less valid for dams emplaced for hydropower production that do not retain large amounts of water. In all settings, it is likely to overestimate the effect of dams relative to other activities or land uses upstream of dams.

Sect S6: Interannual variability in observation-model differences

To assess the influence of interannual variability on unexplained variance in our observation-model comparison, we utilized the years where sampling was conducted by the EPA NRSA (2008, 2009, 2013, 2014, 2018, 2019), because these years are likely to have the most representative and consistent spatial distribution of samples. Every NRSA-sampled year had over 250 observations and represented more than 100 river reaches. No other years exhibited similar spatial representation. We calculated the mean and standard deviation of the observation-model differences and performed a regression on the differences.

The annual mean $\delta^{18}O_{diff}$ and d_{diff} values for NRSA collection years were statistically indistinguishable from the mean differences calculated with the whole dataset (Figure S13). Though all observation-model difference regression slopes were less than 8, and all intercepts were greater than 0, the observation-model difference regression slopes and intercepts were significantly different from one another, and the regression calculated from all available data. This is consistent with the relatively low interannual variability of river observations (standard deviations of less than 2‰, and typically closer to 0.25‰ in $\delta^{18}O$) calculated at sites with at least 4 years of data and 15 data points (Text S3, Figure S3).

One potential source of error in our spatial evaluation of the stable water isotope ratios is the potential for interannual variability in sampling distributions imprinting a temporal climate signal onto our spatial signal. However, the year-to-year similarity in the regression slopes and intercepts, in the context of the relatively low interannual variability, suggests that there is a low likelihood of accidental interpretation of temporal climate patterns as spatial patterns. Likewise, The EPA method is sufficiently standardized (Theobald et al., 2007) that the regression results for each year are unlikely to primarily reflect spatial variability in sampling locations. Instead, the observed interannual variability in slopes and intercepts is more likely to arise from differences in climate and hydrology, which may arise from variability in snowpack isotope ratios and the proportion of surface runoff from rain or snowmelt in the stream relative to groundwater.

Sect S7: Unevaluated sources of variability

We suggest that contributions of irrigation-based recharge to groundwater and subsequent discharge to river flows are likely to be responsible for most of our observation-model differences in arid, irrigation-influenced areas and are a likely source of error in the NWM. However, there is substantial scatter in the data that is independent of the interannual, seasonal, and spatial variability that make an unequivocal demonstration of this mechanism statistically challenging. This scatter may be due to (1) reservoirs and lakes altering the isotopic composition of streamflow in variable and difficult to predict ways (2) changes in the magnitudes of irrigation return flows depending on climate, year, season (Fillo et al., 2021) and irrigation type (Grafton et al., 2018) (3) our ability to estimate irrigation recharge and return flows from available data, (4) evapoconcentration of waterways due to intermittent streamflows and (5) uncertainty in estimating isotopic inputs at an appropriate temporal and spatial scale.

Because lakes exhibit isotopic signals of evaporation (Bowen et al., 2018) relative to other surface waters and precipitation, we hypothesize that reservoirs may contribute to isotopic alteration of surface waters towards evaporation signals. However, our data and key differences between reservoirs and lakes suggest that the relationship between reservoirs and isotope ratios may be more complex (Friedrich et al., 2018). First, reservoirs are typically created by damming existing rivers. Thus, reservoir bathymetry tends to be deeper and narrower than lake bathymetry. This can lead to enhanced stratification, cooler water temperatures, and development of preferential flowpaths of surface water through reservoirs (Deemer et al., 2020, Putman et al., 2024). Irrespective of sampling depth, isotope ratios suggest that deeper, man-made lakes have lower evaporation to inflow ratios than shallow natural lakes (Brooks et al., 2014). Second, dams release water from different levels following temporally variable water management schedules. If a reservoir is stratified, it may mean that the discharged water comes from a more evaporated, warmer surface layer or from a lower, less evaporated, colder layer. The level of the reservoir also shifts over time, so depending on the season or year, the dam may discharge more or less evaporated water depending on the year. Because there are many reservoirs of different sizes, emplaced for different reasons, the effects of reservoirs on isotope ratios of surface waters are likely to be heterogeneous and

unlikely to be efficient at explaining variability in d_{diff}. Instead, smaller natural lakes or wetlands may be more important than large river reservoirs in influencing d_{diff}.

Although we examined the potential for irrigation return flows to contribute evaporated water at lower elevation reaches, it is difficult to estimate the actual contributions of agricultural irrigation recharge to rivers. This difficulty stemmed from ambiguities present in the available datasets, in terms of irrigation timing, irrigation type, water user response to climate variability, spatial and temporal distribution of surface and groundwater withdrawals, water conveyances, estimates of consumptive use (losses to evapotranspiration), variation in groundwater levels, and shallow groundwater flowpaths to surface waters. Relationships between observation-model differences and contribution of agricultural irrigation return flows could improve with increased spatial and temporal granularity of surface and ground water use estimates, improved estimates of consumptive use and improved characterization of groundwater levels.

Although we used the NHDPlus flowlines categorization of streamflow persistence to screen for streams with intermittent flow, these categorizations are difficult (Zipper et al., 2021) and subject to interannual variability. With increasing trends towards intermittent flow in arid regions, and challenges categorizing and assessing intermittent flow (Zipper et al., 2021), it is possible that some of our observations were influenced by evaporation arising from intermittent flows that were not captured by the categorization from NHDPlus.

There are uncertainties associated with the gridded isotope inputs we used to perform our isotope mass balance on the NWM water fluxes. Both the precipitation and groundwater products are estimated from data and interpolated based on some conceptual understanding of controls on stable isotope ratios. Thus, their quality as mixing endmembers in this approach depends on the representativeness of the dataset under conditions similar to those we are testing. For example, interannual variability in snow isotope ratios (Anderson et al., 2016) and variability in the proportions of streamflow from snowmelt runoff, and the seasonal evolution of elevations contributing to runoff, compared with groundwater discharge (Brooks et al., 2021; Wolf et al., 2023), influence the actual isotope ratios of rivers, and may not be captured by our approach. Likewise, long term average summer precipitation isotope ratios may not adequately capture the runoff isotope ratio expected from summer precipitation, as event scale precipitation isotope ratios can exhibit substantial variability (Tulley-Cordova et al., 2021), sometimes on the order of annual variability (Putman et al., 2017).

Figures

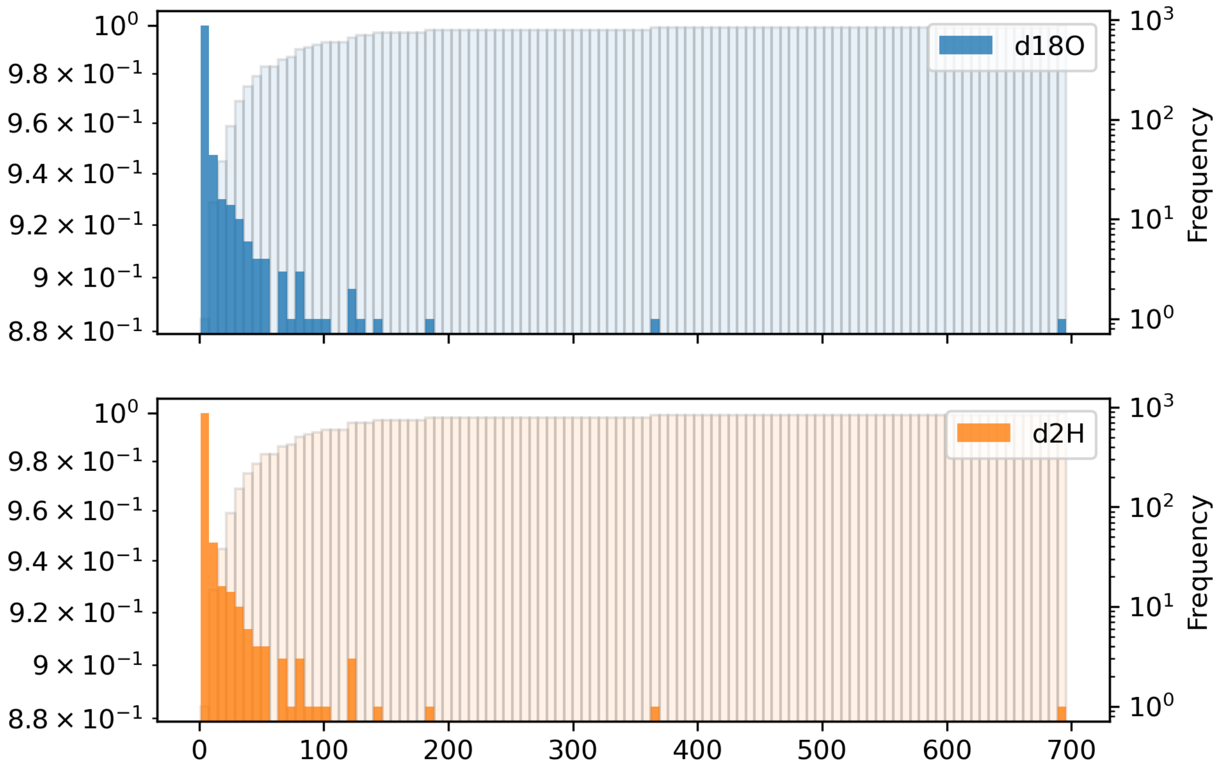

Figure S1: Cumulative distribution function (left axis) and histogram (right axis) showing the distribution of observations per catchment. The cumulative distribution function tells us the percentage of catchments with the bin number or fewer observations – note the log scale. The right axis corresponds to the number of catchments that contained the bin number of observations. The majority (88%) of catchments have fewer than seven observations. However, about 2% of catchments have 100 or more observations.

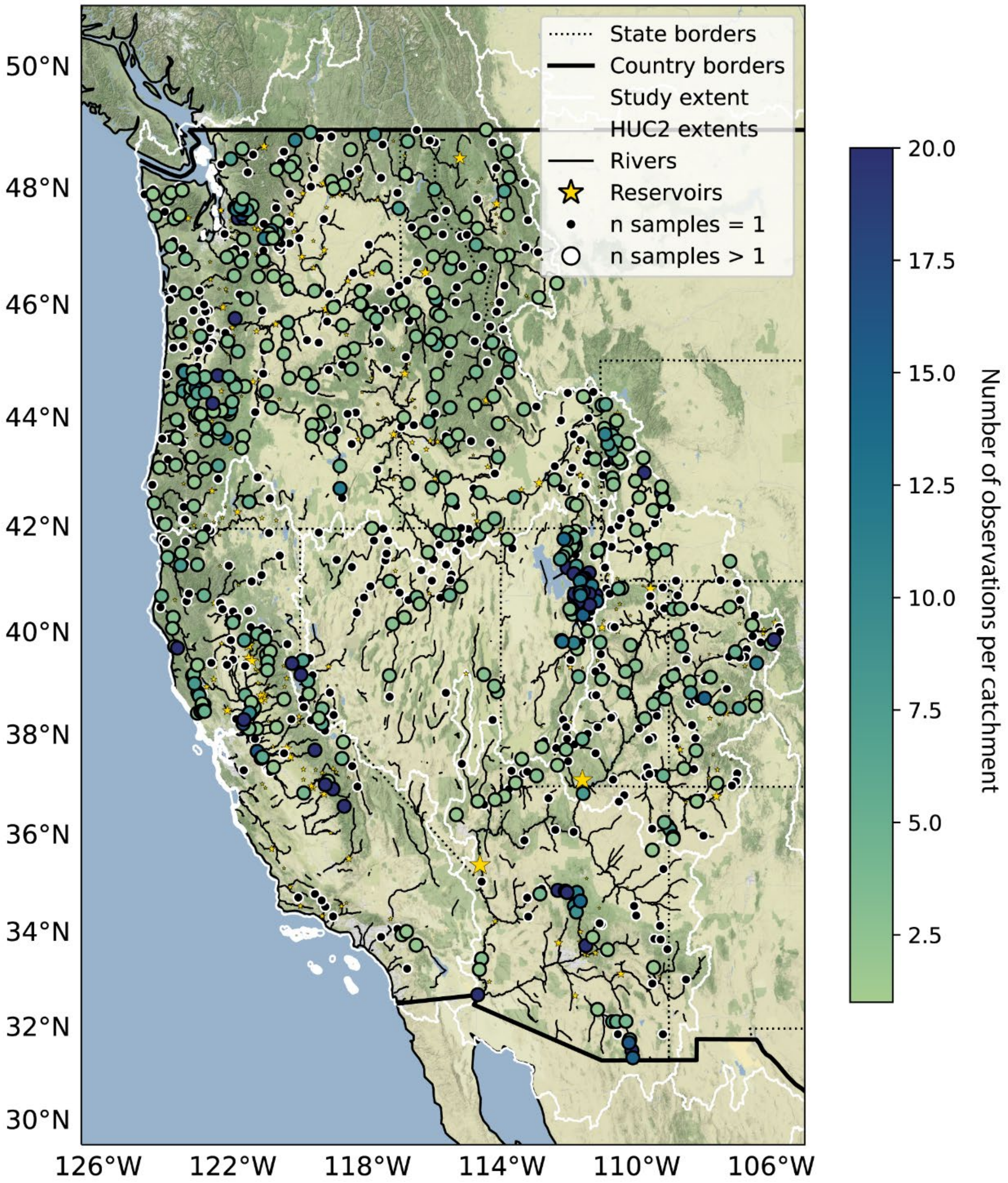

Figure S2: Spatial distribution of the number of observations per catchment. Catchments with a single observation are indicated. Map data is from ©OpenStreetMap contributors 2023, distributed under the Open Data Commons Open Database License (ODbL) v1.0, accessed through Stamen OpenSource Tools (https://stamen.com/open-source/). HUC2 basins from the Watershed Boundary Dataset (U.S. Geological Survey, 2023), and river reaches are modified from the National Hydrography Dataset Plus (U.S. Geological Survey, 2019).

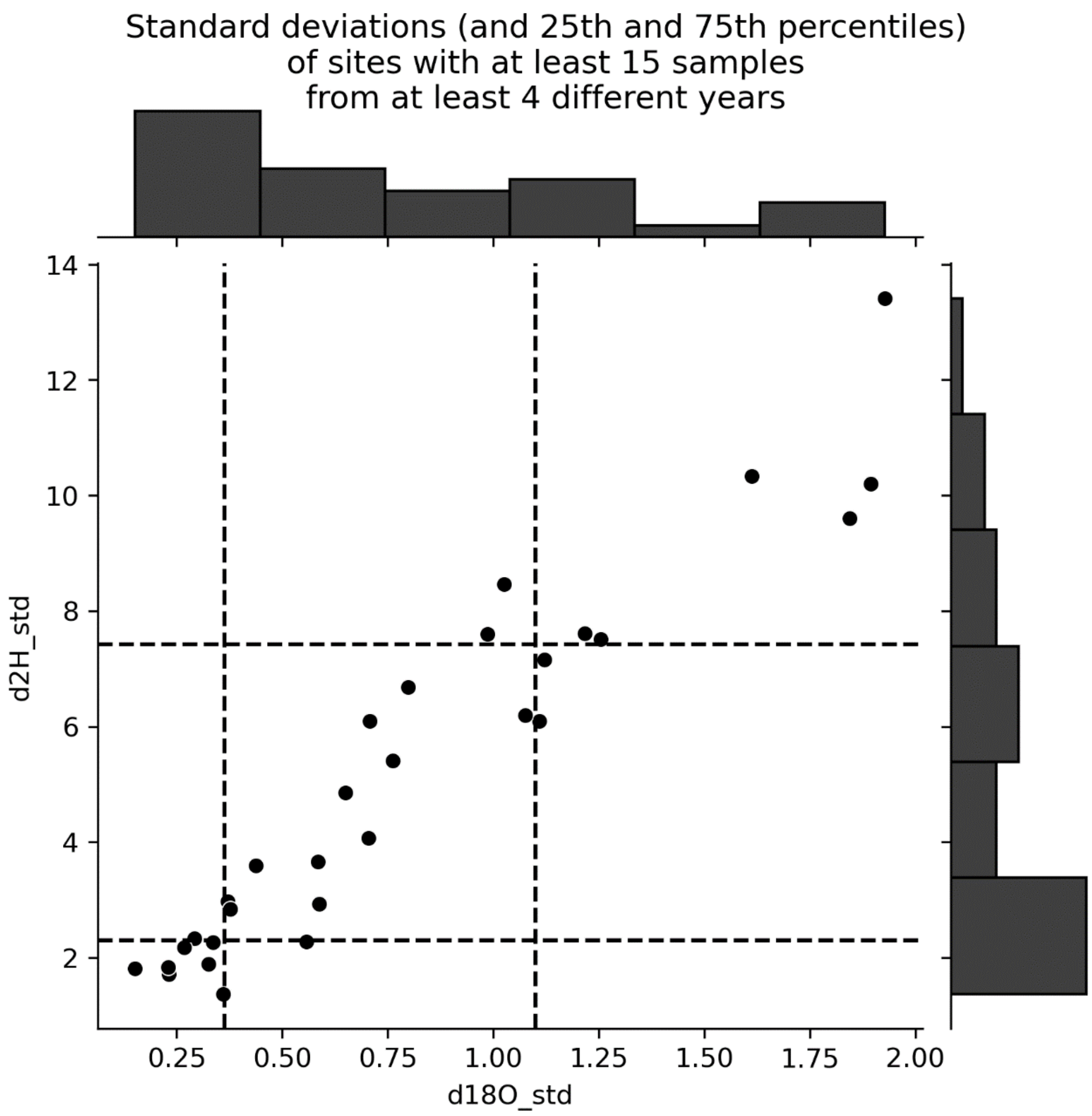

Figure S3: Standard deviations of sites with at least 15 samples from at least 4 different years. The 25th and 75th percentiles are included. We found no correlation between variability and number of samples, stream order, mean annual streamflow, or region.

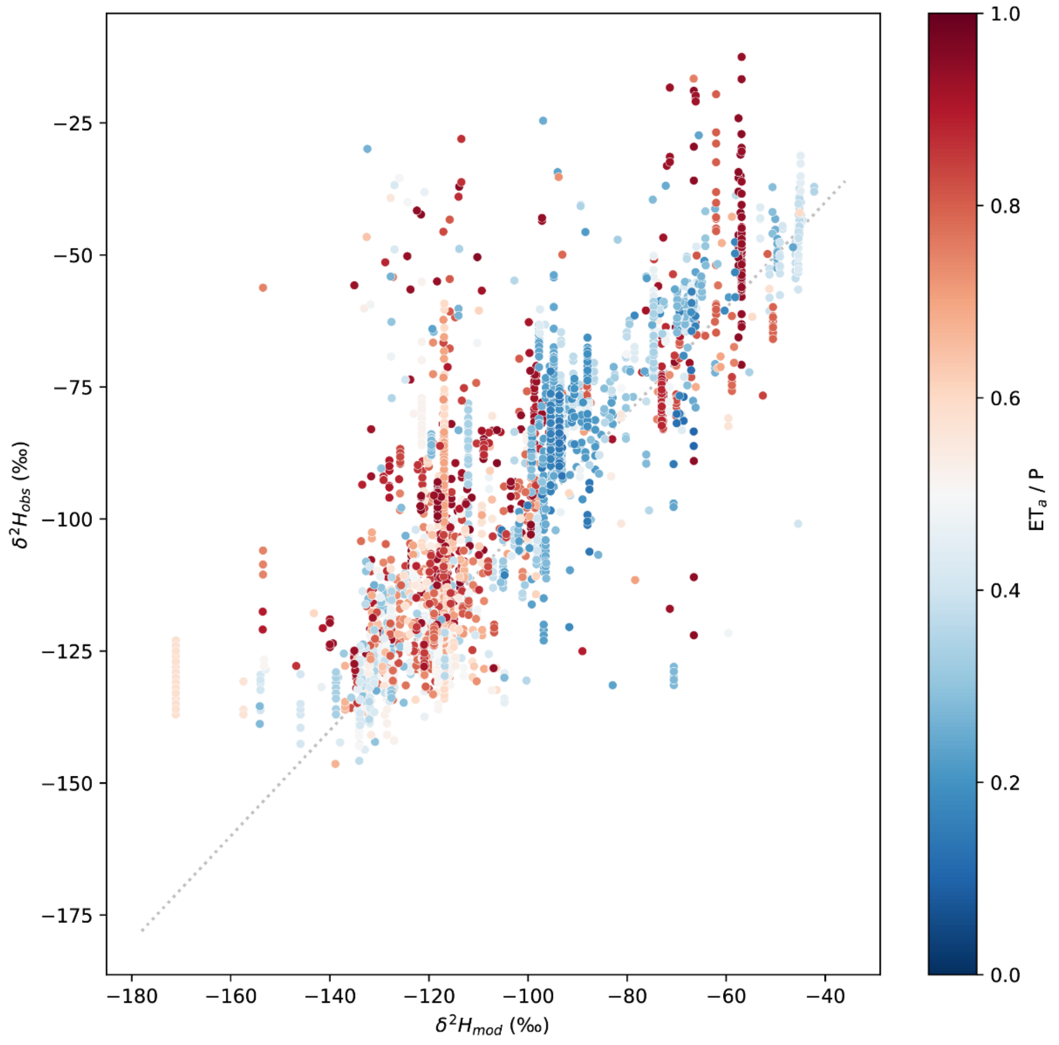

Figure S4: NWM flux-derived Isotope mass balance estimates (mod) compared to all observations (obs) for δ^2H, with dots colored by the ratio of actual evapotranspiration to precipitation. The 1:1 line is plotted for reference. Some catchments may have multiple observations associated with them. Statistics about the model prediction of observation are available from Table 2 in the main text.

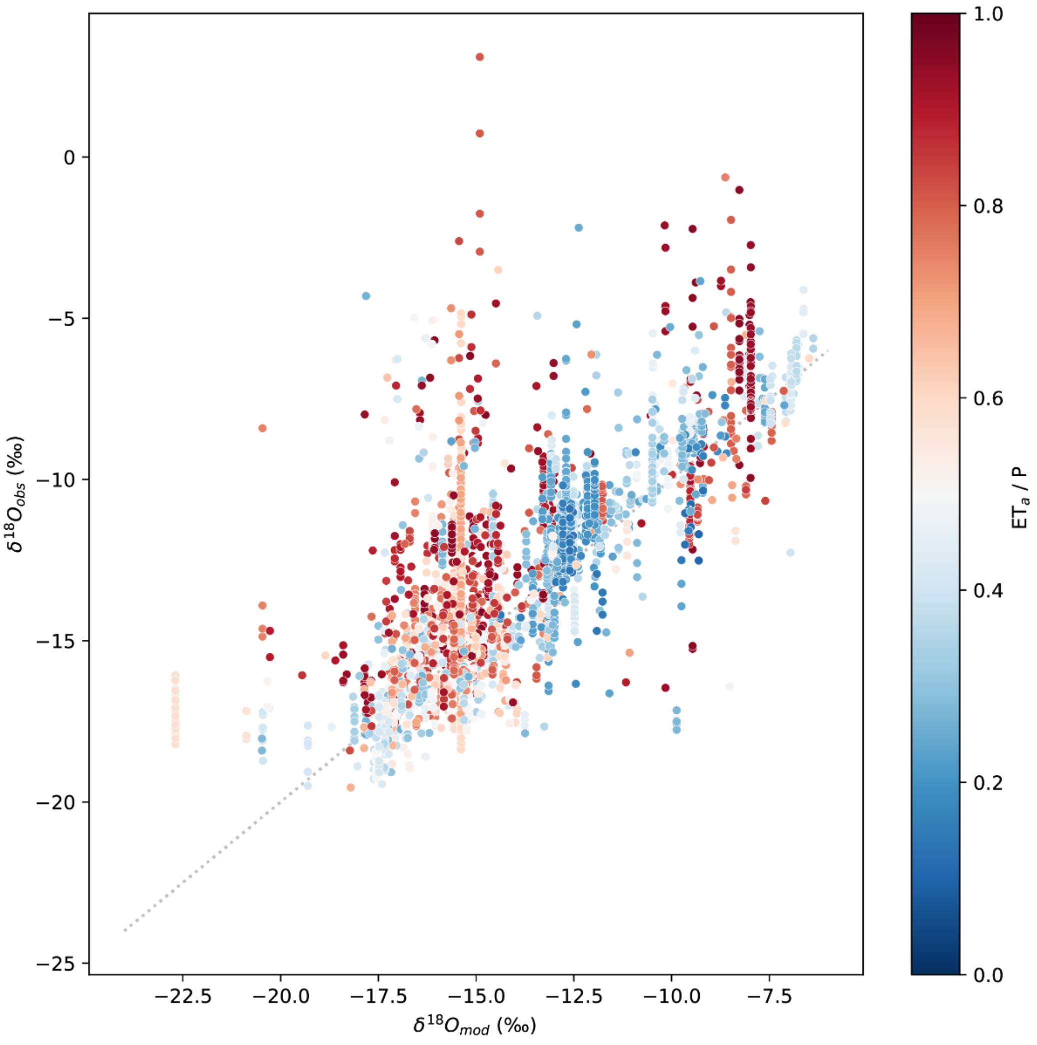

Figure S5: NWM flux-derived Isotope mass balance estimates (mod) estimates compared to all observations (obs) for $\delta^{18}O$, with dots colored by the ratio of actual evapotranspiration to precipitation. The 1:1 line is plotted for reference. Some catchments may have multiple observations associated with them. Statistics about the model prediction of observation are available from Table 2 in the main text.

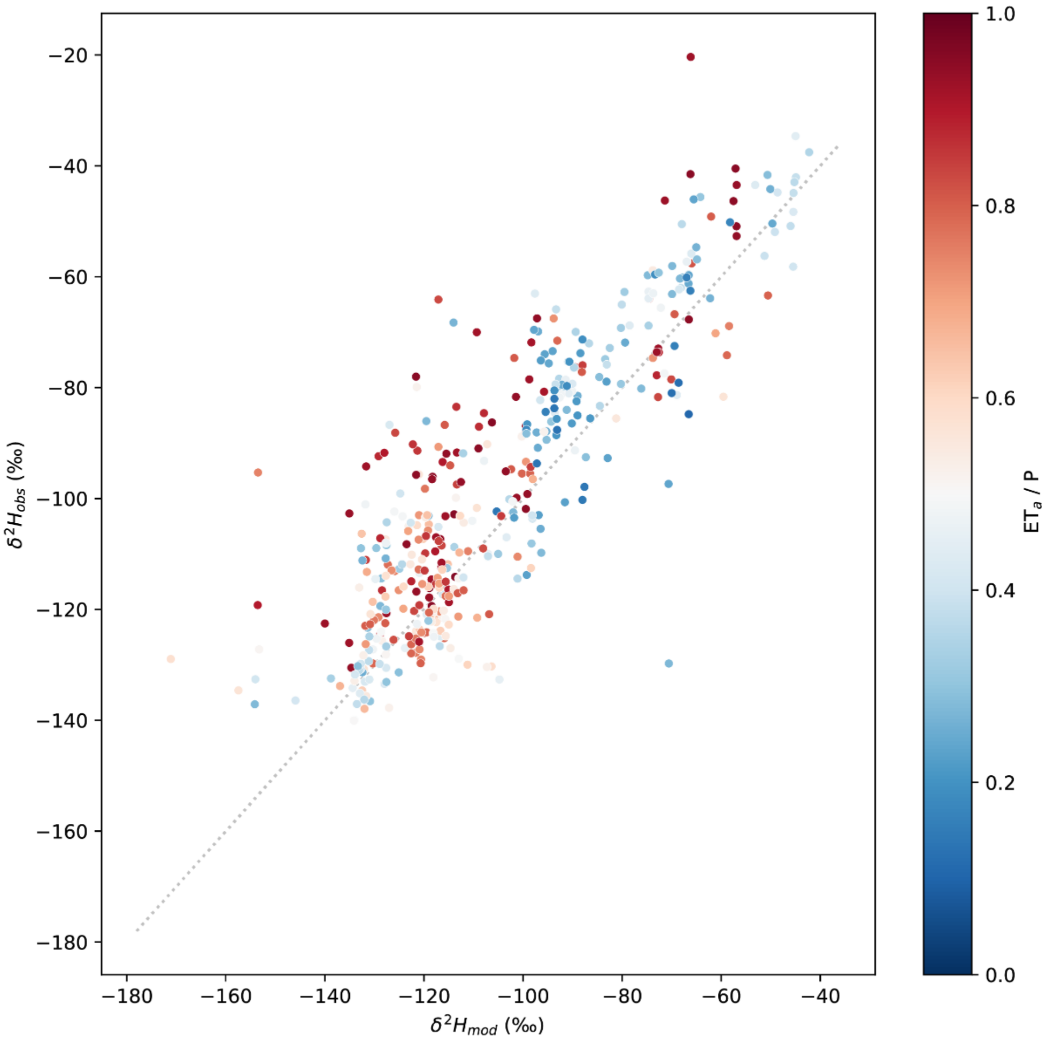

Figure S6: NWM flux-derived Isotope mass balance estimates (mod) estimates compared to mean catchment values of observations (obs) for δ^2H in catchments with at least two observations. Symbol colors indicate the ratio of actual evapotranspiration to precipitation for the catchment. The 1:1 line is plotted for reference. Statistics about the model prediction of observation are available from Table 2 in the main text.

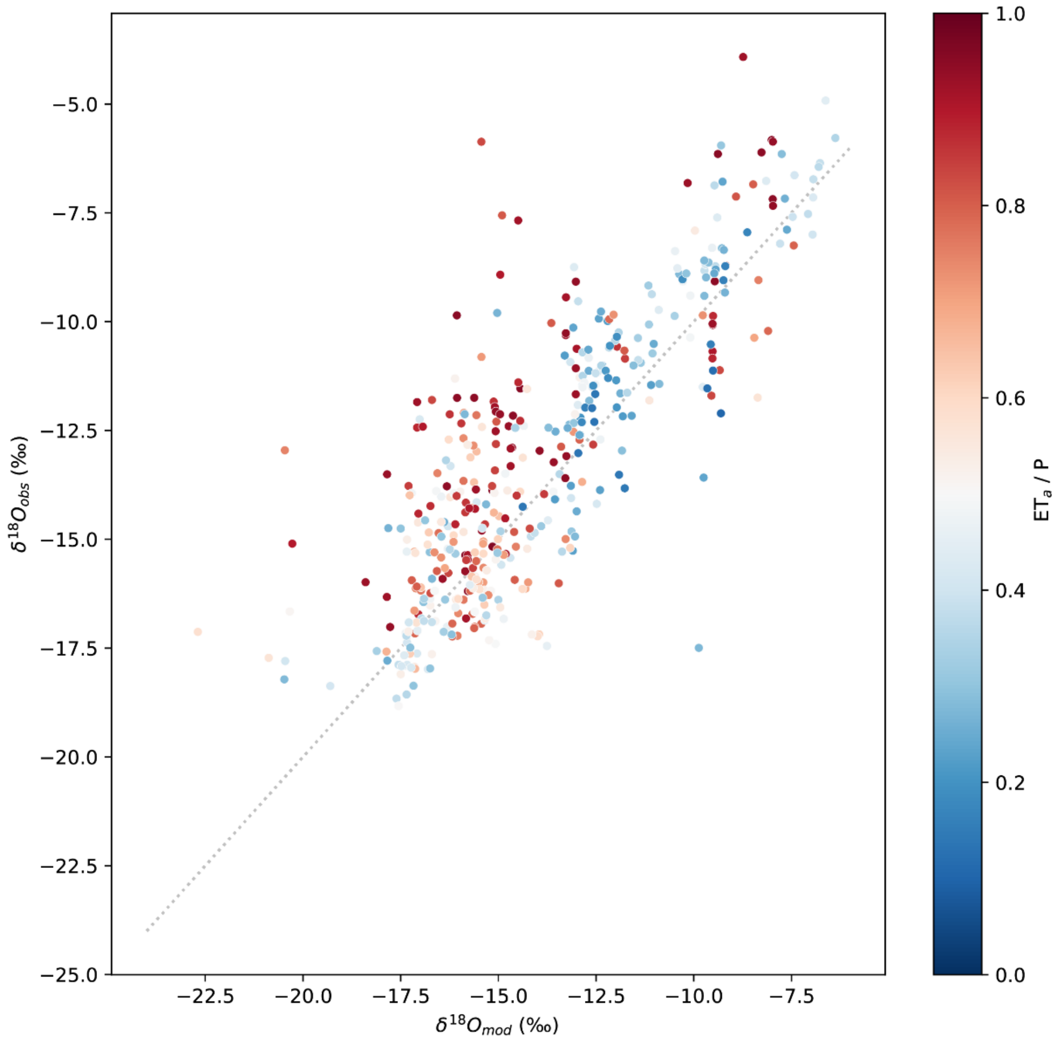

Figure S7: NWM flux-derived Isotope mass balance estimates (mod) estimates compared to mean catchment values of observations (obs) for $\delta^{18}O$ in catchments with at least two observations. Symbol colors indicate the ratio of actual evapotranspiration to precipitation. The 1:1 line is plotted for reference. Statistics about the model prediction of observation are available from Table 2 in the main text.

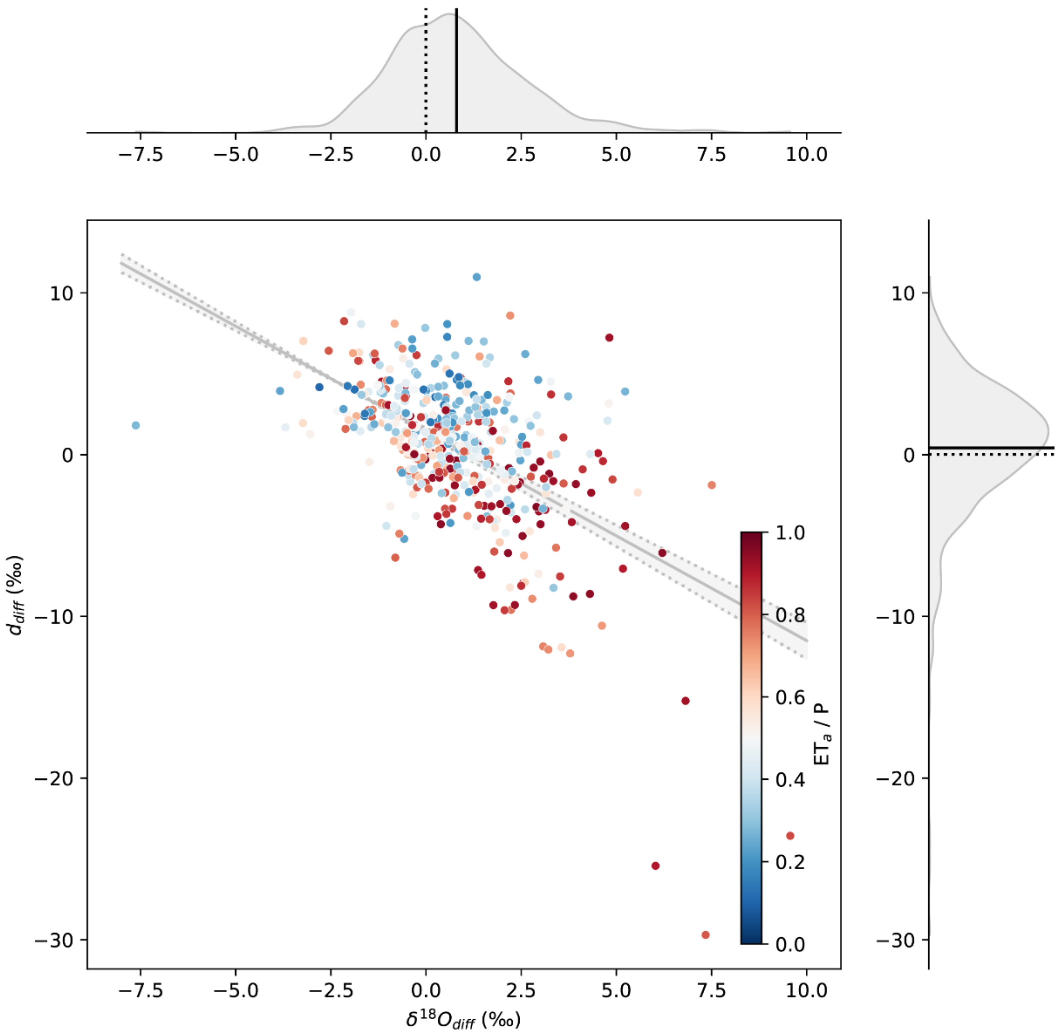

Figure S8: Relationship between $\delta^{18}O_{diff}$ and d_{diff}, with symbols colored by the ratio of actual evapotranspiration to precipitation in each catchment. A linear regression line (gray line) with uncertainty (shaded area with dotted line boundaries) indicates the strength and direction of the relationship between the quantities. Distributions of the quantities are included in the margin axes, and the best fit linear regression and standard error of the regression are also plotted.

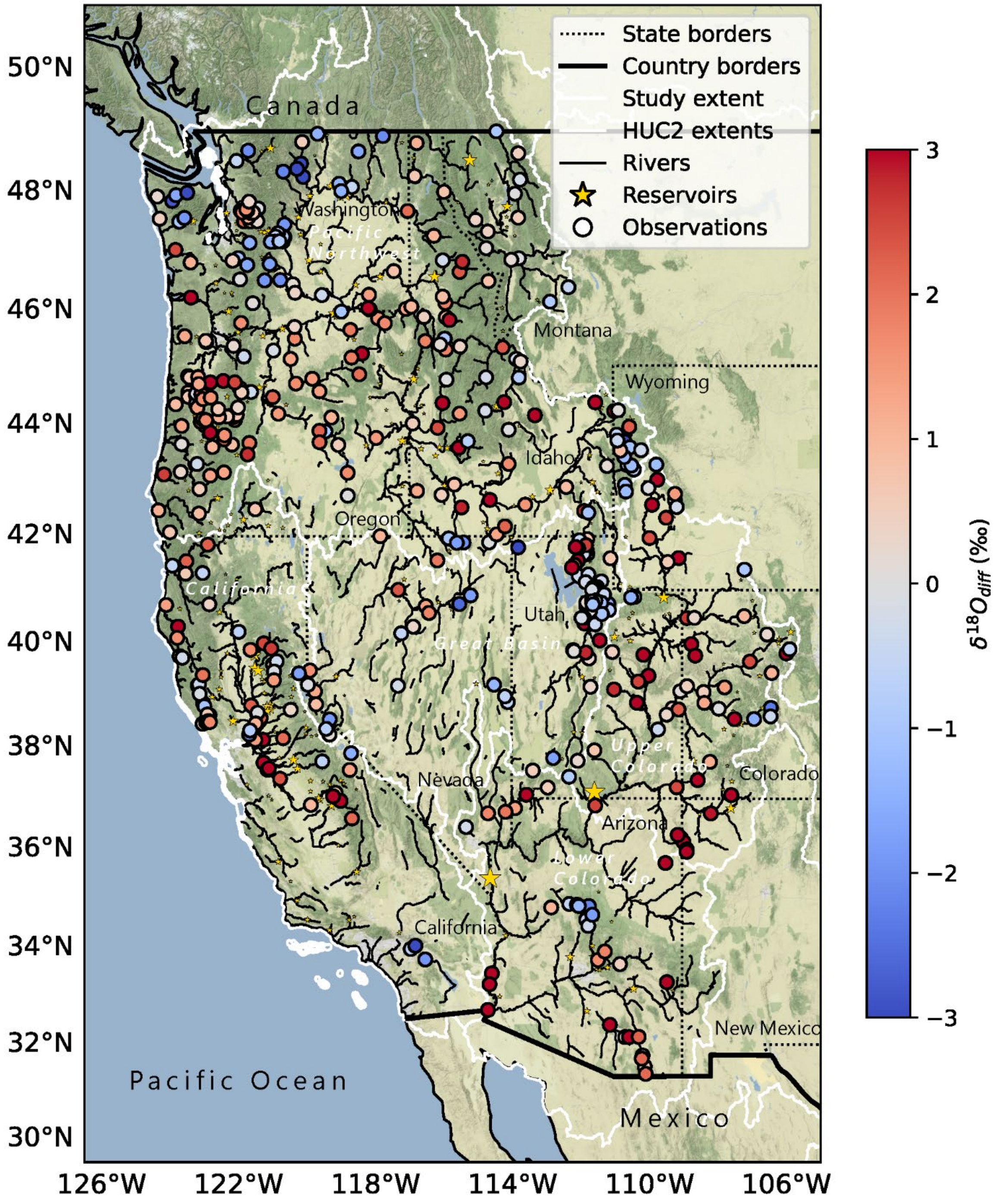

Figure S9: The spatial distribution of mean catchment $\delta^{18}O_{diff}$ in reaches with more than one observation (n=448). Reservoirs are marked by yellow stars, with the star size proportional to the reservoir capacity. Redder shades indicate that observations were more ^{18}O-enriched than the model expected, and bluer shades indicate observations were more ^{18}O-depleted than the model expected. Map data is from ©OpenStreetMap contributors 2023, distributed under the Open Data Commons Open Database License (ODbL) v1.0, accessed through Stamen OpenSource Tools (https://stamen.com/open-source/). HUC2 basins from the Watershed Boundary Dataset (U.S. Geological Survey, 2023), and river reaches are modified from the National Hydrography Dataset Plus (U.S. Geological Survey, 2019).

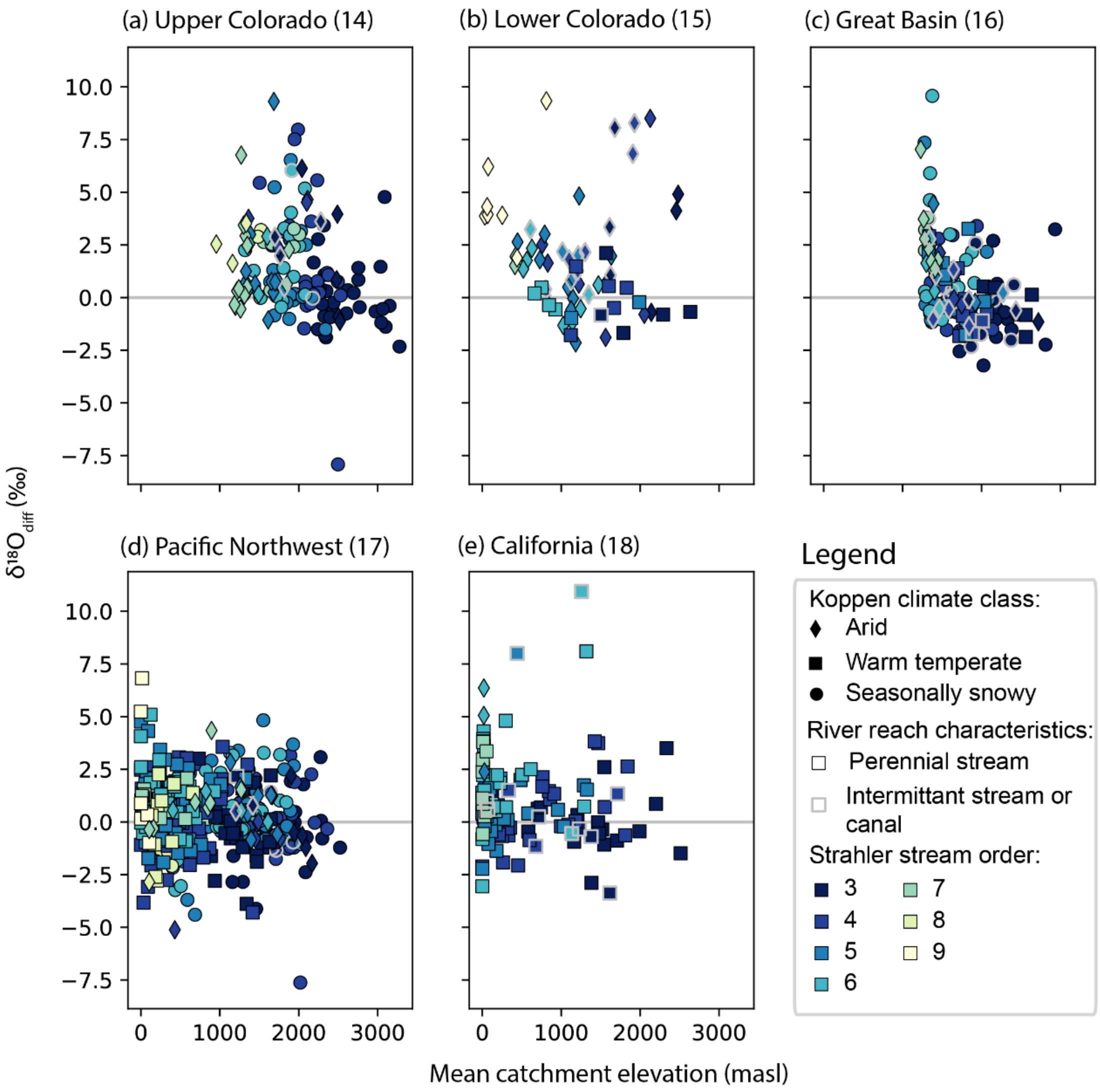

Figure S10: Relationship of elevation, Strahler stream order (U.S. Geological Survey, 2019), and Köppen climate classification (Rubel and Kottek, 2010), and stream persistence to $\delta^{18}O_{diff}$ in each basin. We observe lower $\delta^{18}O_{diff}$ with perennial, lower order streams at middle and higher elevations in each basin. Higher $\delta^{18}O_{diff}$ is associated with higher order streams at lower elevations in each basin. This effect was greater in catchments classified as arid or seasonally snowy compared to those classified as warm temperate. This pattern was generally true in each basin, irrespective of the absolute elevation or stream order, suggesting the importance of accumulated effects within a basin on $\delta^{18}O_{diff}$.

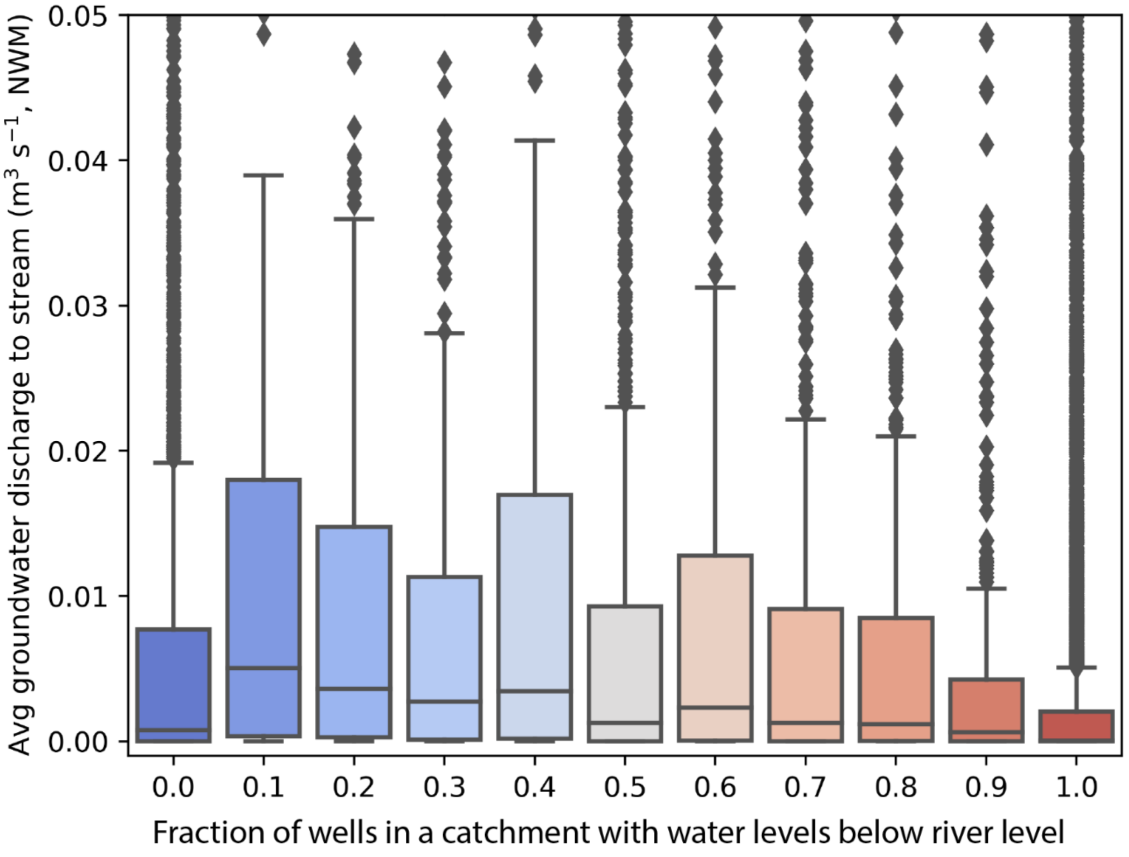

Figure S11: Relationship between the fraction of well water levels that are below the river level (Jasechko et al., 2021) and the average summer groundwater discharge to a stream (National Atmospheric and Oceanic Administration, 2022). The fractions of wells with water levels below river water level have been binned for simplification of displaying data. There is a statistically significant, though weak relationship (R2 = 0.007) between the quantities.

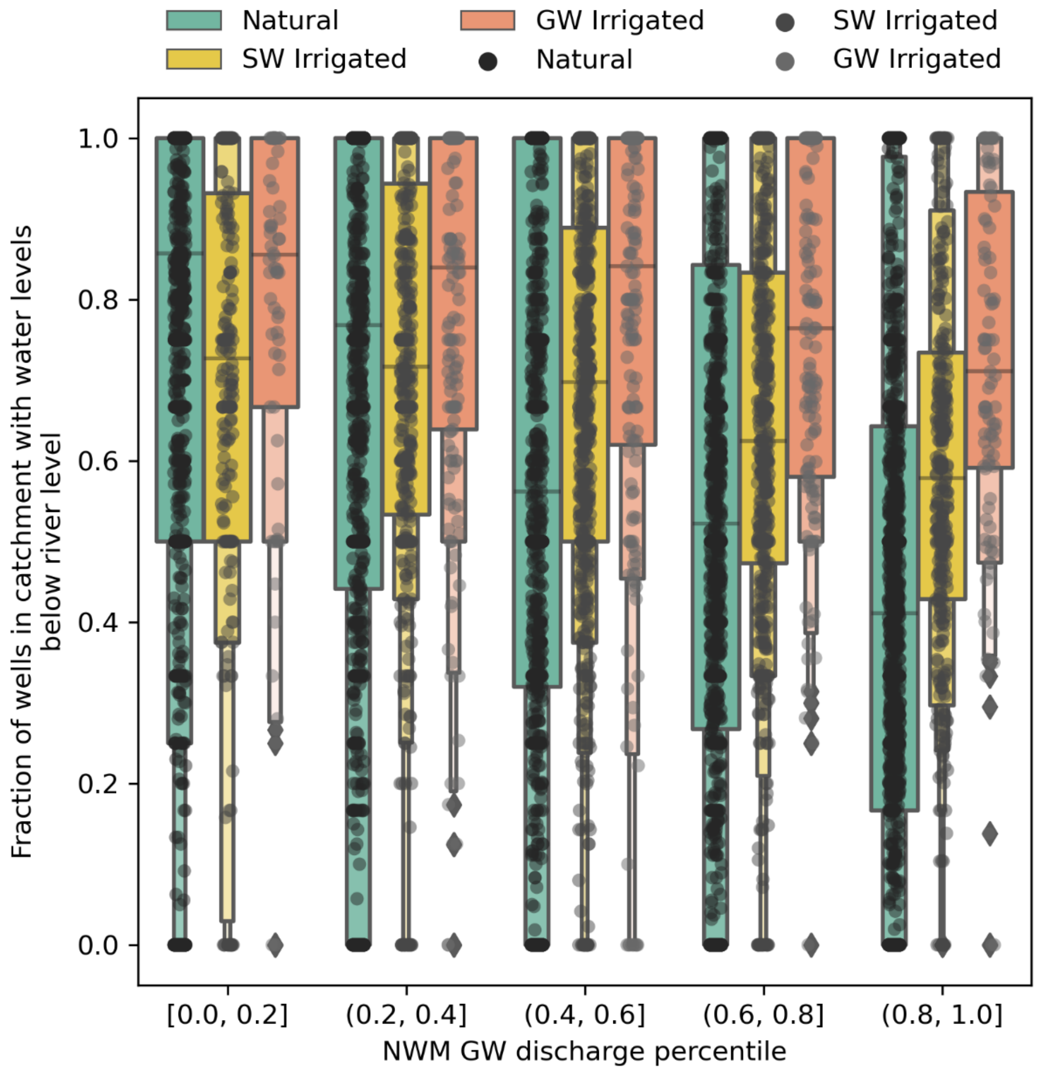

Figure S12: Relationship between groundwater discharge in NWM (National Atmospheric and Oceanic Administration, 2022) and the Jasechko et al (2021) estimate of the number of well water levels that are below river level, differentiated by presence of agricultural irrigation, and the water source for that irrigation. Groundwater (GW) and Surface water (SW) are the two categories of irrigation water sources. The boxenplots for each groundwater discharge percentile indicate the distribution quantiles.

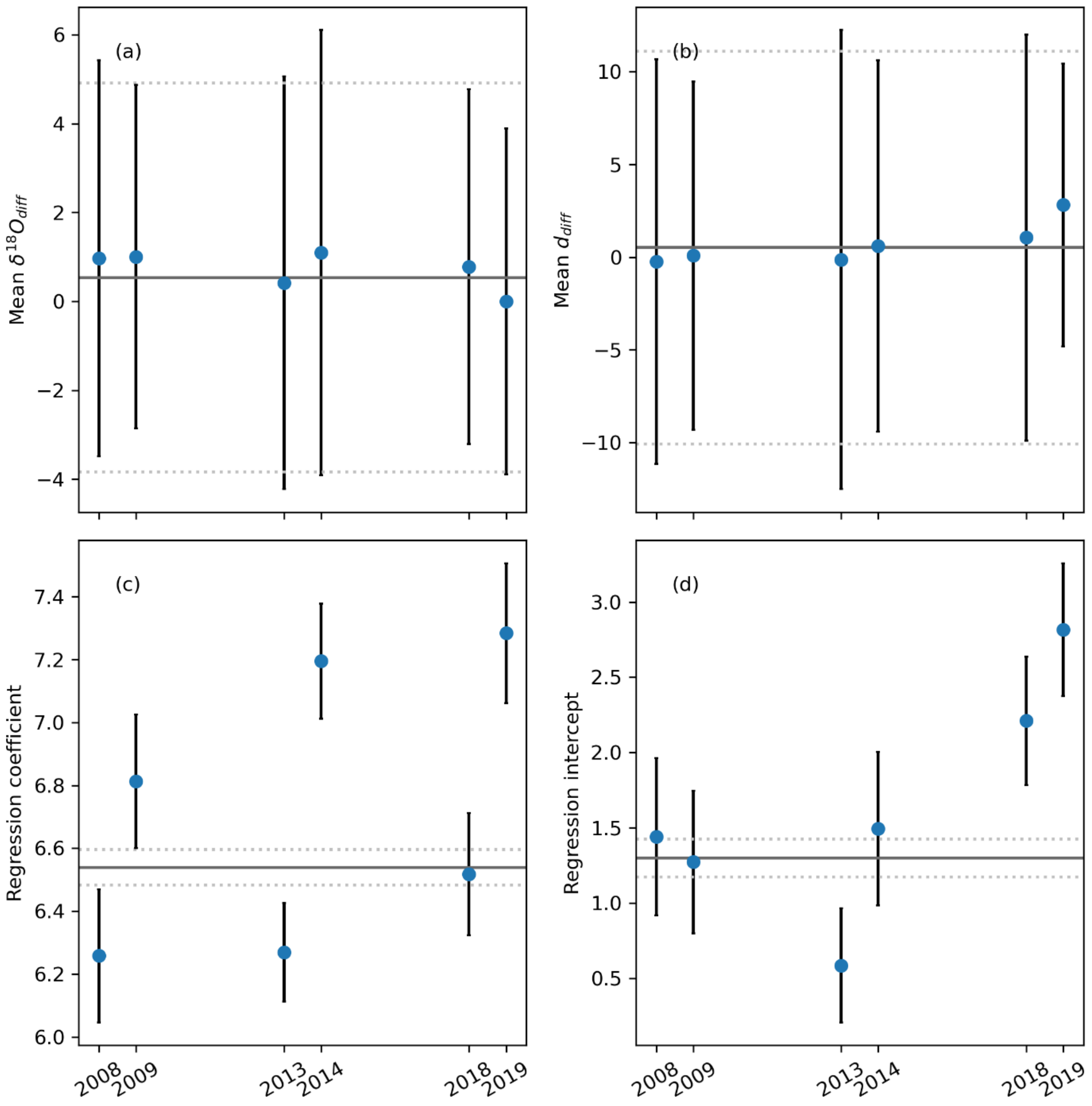

Figure S13: Interannual variability in observation-model comparisons using data from years with at least 250 samples in 100 different reaches (NRSA years). (a) The annual mean (blue points) and 95% confidence intervals (solid vertical lines) of $\delta^{18}O_{diff}$ compared to the mean (solid horizontal line) and 95% confidence intervals (dotted horizontal lines) of all data. (b) The annual mean and 95% confidence intervals of d_{diff} compared to the mean and 95% confidence intervals of all data. (c) The slope of δ^2H_{diff} ~ $\delta^{18}O_{diff}$ and 95% confidence intervals compared to the slope and 95% confidence intervals of calculated from all data. (d) The regression intercept of δ^2H_{diff} ~ $\delta^{18}O_{diff}$ and 95% confidence intervals compared to the regression intercept and 95% confidence intervals of calculated from all data. In all subplots, the all-data mean and 95% confidence interval are plotted for reference.

Any use of trade, firm, or product names is for descriptive purposes only and does not imply endorsement by the U.S. Government.

References

Abatzoglou, J. T. (2013). Development of gridded surface meteorological data for ecological applications and modelling. Int. J. Climatol., 33, 121–131. doi: https://doi.org/10.1002/joc.3413

Anderson, L., Berkelhammer, M., & Mast, M. A. (2016). Isotopes in North American Rocky Mountain Snowpack 1993–2014. Quaternary Science Reviews, 131, 262-273. doi: https://doi.org/10.1016/j.quascirev.2015.03.023

Army Corps of Engineers: Federal Emergency Management Agency, U. A. C. (n.d.). National inventory of dams. Retrieved from https://nid.sec.usace.army.mil (Data product, accessed 10-2022)

Bowen, G. J., et al. (2018). Inferring the source of evaporated waters using stable H and O isotopes. Oecologia, 187 (4), 1025-1039. doi: 10.1007/s00442-018-4192-5

Brooks, J. R., et al. (2014). Stable isotope estimates of evaporation: Inflow and water residence time for lakes across the United States as a tool for national lake water quality assessments. Limnology and Oceanography, 59 (6), 2150-2165. doi: https://doi.org/10.4319/lo.2014.59.6.2150

Brooks, P. D., et al. (2021). Groundwater-mediated memory of past climate controls water yield in snowmelt-dominated catchments. Water Resources Research, 57 (10), e2021WR030605. doi: https://doi.org/10.1029/2021WR030605

Deemer, B. R., Stets, E. G., & Yackulic, C. B. (2020). Calcite precipitation in Lake Powell reduces alkalinity and total salt loading to the Lower Colorado River Basin. Limnology and Oceanography, 65 (7), 1439-1455. doi: https://doi.org/10.1002/lno.11399

Dewitz, J., and U.S. Geological Survey, 2021, National Land Cover Database (NLCD) 2019 Products (ver. 2.0, June 2021): U.S. Geological Survey data release, doi: https://doi.org/10.5066/P9KZCM54.

Dieter, C.A., et al. (2018), Estimated use of water in the United States in 2015: U.S. Geological Survey Circular 1441, 65 p., https://doi.org/10.3133/cir1441. [Supersedes USGS Open-File Report 2017–1131.]

Fillo, N. K., Bhaskar, A. S., & Jefferson, A. J. (2021). Lawn irrigation contributions to semi-arid urban baseflow based on water-stable isotopes. Water Resources Research, 57 (8), e2020WR028777. doi: https://doi.org/10.1029/2020WR028777

Friedrich, K., et al., (2018). Reservoir evaporation in the western United States: Current science, challenges, and future needs. Bulletin of the American Meteorological Society, 99 (1), 167 - 187. doi: https://doi.org/10.1175/BAMS-D-15-00224.1

Grafton, R. Q., et al., (2018). The paradox of irrigation efficiency. Science, 361 (6404), 748-750. doi: 10.1126/science.aat9314

Hutson, S. S. et al. (2004). Estimated use of water in the United States in 2000 (Vol. 1268). US Geological Survey Circular 1268. https://pubs.usgs.gov/circ/2004/circ1268/

Jasechko, S., et al. (2014). The pronounced seasonality of global groundwater recharge. Water Resources Research, 50 (11), 8845-8867. doi: https://doi.org/10.1002/2014WR015809

Jasechko, S., Seybold, H., & Perrone, D. e. a. (2021). Widespread potential loss of streamflow into underlying aquifers across the USA. Nature, 591 , 391–395. doi: https://doi.org/10.1038/s41586-021-03311-x

Jordahl, K. et al.(2020). geopandas/geopandas: v0.8.1 (Version v0.8.1). Zenodo. doi: http://doi.org/10.5281/zenodo.3946761

Kenny, J.F et al. (2009) Estimated use of water in the United States in 2005: U.S. Geological Survey Circular 1344, 52 p. https://pubs.usgs.gov/circ/1344/

Kirchner, J. W. (2016). Aggregation in environmental systems – part 2: Catchment mean transit times and young water fractions under hydrologic nonstationarity. Hydrology and Earth System Sciences, 20 (1), 299–328. doi: https://doi.org/10.5194/hess-20-299-2016

Maupin, M.A., et al. (2014) Estimated use of water in the United States in 2010: U.S. Geological Survey Circular 1405, 56 p., https://dx.doi.org/10.3133/cir1405.

National Oceanographic and Atmospheric Administration (2022) National Water Model CONUS Retrospective Dataset, https://registry.opendata.aws/nwm-archive, data accessed in 2022

Putman, A. L., Feng, X., Sonder, L. J., & Posmentier, E. S. (2017). Annual variation in event-scale precipitation δ^2H at Barrow, AK, reflects vapor source region. Atmospheric Chemistry and Physics, 17, 4627–4639. doi: https://doi.org/10.5194/acp-17-4627-2017

Putman, A. L., Fiorella, R. P., Bowen, G. J., & Cai, Z. (2019). A global perspective on local meteoric water lines: Meta-analytic insight into fundamental controls and practical constraints. Water Resources Research, 55 (8), 6896–6910. doi: https://doi.org/10.1029/2019WR025181

Putman, A. L., McIlwain, H. E., Rumsey, C. A., & Marston, T. M. (2024). Low flows from drought and water use reduced total dissolved solids fluxes in the Lower Colorado River Basin between 1976 to 2008. Journal of Hydrology: Regional Studies, 52, 101673. https://doi.org/10.1016/j.ejrh.2024.101673

Reddy, J., Longley, P. C., McDonnell, M. C., Katoski, M. P., Miller, O. L., and Putman, A. (2022) Hydrogen and oxygen stable isotope mass balance evaluation of the National Water Model (v2.1) streamflow, runoff and groundwater flows, https://doi.org/10.5066/P9NOD5ES, US Geological Survey Data Release

Rubel, F., & Kottek, M. (2010). Observed and projected climate shifts 1901-2100 depicted by world maps of the Köppen-Geiger climate classification. Meteorologische Zeitschrift, 19(2), 135. Doi: https://doi.org/10.1127/0941-2948/2010/0430

Theobald, D. M., et al., (2007) Using GIS to Generate Spatially Balanced Random Survey Designs for Natural Resource Applications, Environmental Management, 40, 134–146, https://doi.org/10.1007/s00267-005-0199-x

Tulley-Cordova, C. L., Putman, A. L., & Bowen, G. J. (2021). Stable isotopes in precipitation and meteoric water: Sourcing and tracing the North American Monsoon in Arizona, New Mexico, and Utah. Water Resources Research, 57 (12), e2021WR030039. doi: https://doi.org/10.1029/2021WR030039

U.S. Geological Survey (2023) National Geospatial Technical Operations Center: Watershed Boundary Dataset (WBD) - USGS National Map Downloadable Data Collection, https://www.sciencebase.gov/catalog/item/51361e87e4b03b8ec4025c22#:~:text=Citation,Data%20Collection%3A%20U.S.%20Geological%20Survey., ScienceBase Data Release, 2023.

U.S. Geological Survey (2019) National Hydrography Dataset (ver. 2.1), https://www.usgs.gov/national-hydrography/access-national-hydrography-products, accessed Oct, 2021, 2019

Wolf, M. A., et al., (2023). Multi-year controls on groundwater storage in seasonally snow-covered headwater catchments. Water Resources Research, 59 (6), e2022WR033394. doi: https://doi.org/10.1029/2022WR033394

Zipper, S. C., et al., (2021). Pervasive changes in stream intermittency across the United States. Environmental Research Letters, 16 (8), 084033. doi: https://doi.org/1241 10.1088/1748-9326/ac14ec
